# Trends in disease burden of chronic myeloid leukemia at the global, regional, and national levels: a population-based epidemiologic study

**DOI:** 10.1186/s40164-020-00185-z

**Published:** 2020-11-03

**Authors:** Liqing Ning, Chuanyu Hu, Pingfan Lu, Yimei Que, Xiaojian Zhu, Dengju Li

**Affiliations:** 1 Department of Hematology, Tongji Hospital, Tongji Medical College, Huazhong University of Science and Technology, Wuhan, 430030 China; 2 Department of Stomatology, Tongji Hospital, Tongji Medical College, Huazhong University of Science and Technology, Wuhan, 430030 China

**Keywords:** Chronic myeloid leukemia, Global burden, Cancer epidemiology, Incidence, Deaths

## Abstract

**Background:**

Outcomes of chronic myeloid leukemia (CML) has been improved dramatically in the past two decades, but survival levels of CML patients varied in regions. Comprehensive epidemiological research is necessary to evaluate the global burden of CML.

**Methods:**

All data used in our study came from the Global Burden of Disease (GBD) study 2017. Incidence cases, death cases, disability-adjusted life-years (DALYs), and its corresponding age-standardized rate between 1990 to 2017 were used to describe the distribution of CML burden, according to age, sex, social-demographic index (SDI), and countries. Data about attributable risk factors contributing to CML deaths and DALYs were also extracted and analyzed.

**Results:**

Globally, the disease burden of CML gradually decreased from 1990 to 2017. Higher SDI countries achieved a remarkable effect on diminishing the CML burden. Conversely, due to population growth, the incidence cases, death cases, and DALYs of CML in lower SDI quintiles showed an upward trend. India had the most incidence cases and death cases of CML in the world. Additionally, smoking was the most significant attributable risk factor contributing to CML deaths and DALYs, followed by high body mass index.

**Conclusion:**

The disease burden of CML decreased globally, especially in higher SDI countries in the past 28 years. The increasing incidence cases and death cases were mainly observed in lower SDI countries. Additionally, strategies to control modifiable risk factors such as smoking and high body mass index might be useful in diminishing mortality and DALYs.

## Background

Chronic myeloid leukemia (CML) is a clonal hematopoietic stem cell disorder with characteristic Philadelphia chromosome, which leads to an excessive burden of myeloid cells in patients [1]. CML is an age-related neoplasm that commonly happened in older people. The median age at diagnosis was estimated to be 60 years in western countries, but the age at diagnosis in Africa and Asia was about ten years younger [2, 3]. CML is the first cancer with specific genotype knowledge, which led to a rationally therapeutic schedule [4]. Imatinib, a tyrosine kinase inhibitor (TKI), was approved by the FDA to treat CML in 2001 and revolutionized the treatment pattern of this disease [5, 6]. Indeed, thanks to TKI-based treatment, CML's status switched from a lethal disease to a chronic disease, especially for patients in the chronic phase [7, 8]. The apparent improvement in the survival of CML patients mainly occurred in high-income countries like the United States, France, and Japan [9–11]. The disease burden of CML distinctly varies in different countries due to diverse opportunities for early-stage screening, novel drugs and medical resources [12, 13]. The worldwide disease burden of CML is worth evaluating, which can help us better understand the specific impact of this disease on public health.

The Global Burden of Disease (GBD) study 2017 contains epidemiologic data about 354 diseases across 195 countries and territories, providing an opportunity to understand the worldwide trends in the disease burden of CML [13]. Accordingly, we described the incidence, deaths, disability-adjusted life-years (DALYs), and its attributable risk factors of CML by age, sex, geographical regions, social-development index (SDI) on specific CML data from the (GBD) study 2017.

## Materials and methods

### Data source

We derived data from the public database GBD study 2017. Previous studies had carefully described the guidelines and methodology of the GBD study [14, 15]. Besides, the GBD study estimates the levels and trends of 84 attributable risk factors associated with disease burden based on the comparative risk assessment framework [16]. We extracted data from Global Health Data Exchange query tool (https://ghdx.healthdata.org/gbd-re-sults-tool). Data regarding annual CML incidence, deaths, DALYs, and attributable risk factors related to CML burden, and information about age, regions, sex, and countries were collected. GBD study 2017 divided the world into five social-demographic index (SDI) quintiles (high, high-middle, middle, low-middle, low) and 21 geographical regions (Tables 1, 2 and 3).

**Table 1:** The incidence cases and ASIR of CML in 1990 and 2017, and its temporal trends from 1990 to 2017

	Incident cases no. *10^2^ (95% UI)		Change in absolute number (%)	ASIR per 100,000 No. (95% UI)		1990–2017 EAPCs No. (95% CI)
	1990	2017		1990	2017	
Overall	317.52 (295.9–340.66)	341.79 (315.16–367.14)	7.64	0.75 (0.71–0.8)	0.43 (0.4–0.46)	-2.4 (− 2.53 to − 2.26)
Sex						
Male	165.57 (153.46–177.23)	191.45 (173.2–208.22)	15.64	0.86 (0.8–0.92)	0.52 (0.47–0.57)	− 2.17 (− 2.29 to − 2.05)
Female	151.96 (135.82–171.91)	150.33 (130.96–170.43)	− 1.07	0.67 (0.61–0.75)	0.36 (0.31–0.41)	− 2.7 (− 2.85 to − 2.55)
Socio- demographic index						
High SDI	163.42 (157.15–169.51)	106.4 (100.95–112.18)	− 34.89	1.34 (1.29–1.38)	0.53 (0.51–0.56)	− 4 (− 4.23 to − 3.78)
High-middle SDI	49.58 (43.76–55.26)	57.35 (52.42–62.39)	15.69	0.49 (0.43–0.54)	0.33 (0.3–0.36)	− 1.77 (− 1.95 to − 1.6)
Middle SDI	38.31 (34.17–44.49)	67.71 (58.04–72.69)	76.73	0.33 (0.3–0.39)	0.3 (0.26–0.32)	− 0.56 (− 0.66 to − 0.45)
Low-middle SDI	32.8 (28.42–41.59)	56.67 (49.19–65.98)	72.79	0.48 (0.42–0.61)	0.43 (0.37–0.5)	− 0.48 (− 0.6 to − 0.36)
Low SDI	32.8 (25.9–41.69)	52.85 (44.44–59.95)	61.14	0.81 (0.67–1.01)	0.65 (0.55–0.74)	− 0.93 (− 0.97 to − 0.88)
Region						
Andean Latin America	0.87 (0.73–1)	2.08 (1.69–2.39)	138.66	0.34 (0.28–0.38)	0.36 (0.29–0.42)	0.46 (0.32–0.6)
Australasia	3.87 (3.36–4.44)	3.58 (2.95–4.32)	− 7.43	1.64 (1.43–1.87)	0.83 (0.68–1)	− 3.35 (− 3.66 to − 3.03)
Caribbean	2.64 (2.41–2.91)	3.07 (2.73–3.48)	16.28	0.93 (0.85–1.02)	0.61 (0.54–0.69)	− 1.7 (− 1.84 to − 1.57)
Central Asia	2.52 (2.16–2.88)	2.74 (2.41–3.07)	8.73	0.45 (0.39–0.51)	0.32 (0.28–0.36)	− 1.34 (− 1.57 to − 1.11)
Central Europe	11.11 (10.48–11.77)	6.82 (6.42–7.27)	− 38.62	0.77 (0.73–0.82)	0.37 (0.35–0.4)	− 2.87 (− 3.14 to − 2.6)
Central Latin America	6.98 (6.69–7.4)	11.96 (11.22–12.85)	71.35	0.66 (0.64–0.7)	0.49 (0.46–0.53)	− 1.47 (− 1.7 to − 1.23)
Central Sub-Saharan Africa	1.49 (0.99–1.92)	3.61 (2.64–4.51)	142.15	0.51 (0.35–0.64)	0.57 (0.42–0.72)	0.58 (0.47 to 0.69)
East Asia	22.64 (17.43–28.02)	32.71 (27.97–37.64)	44.49	0.19 (0.16–0.24)	0.17 (0.15–0.19)	− 0.99 (− 1.21 to − 0.76)
Eastern Europe	18.01 (15.98–20.72)	18.51 (16.46–20.67)	2.77	0.66 (0.59–0.75)	0.61 (0.54–0.69)	− 0.55 (− 0.86 to − 0.25)
Eastern Sub-Saharan Africa	11.1 (8.15–15.09)	16.39 (12.56–19.89)	47.6	1.11 (0.86–1.49)	0.81 (0.63–0.98)	− 1.41 (− 1.51 to − 1.31)
High-income Asia Pacific	17.08 (15.68–18.57)	12.81 (11.33–14.46)	− 25.03	0.87 (0.79–0.94)	0.39 (0.34–0.44)	− 3.2 (− 3.37 to − 3.02)
High-income North America	34.1 (32.98–35.37)	20.55 (19.44–21.81)	− 39.75	1.02 (0.98–1.06)	0.4 (0.37–0.43)	− 4.28 (− 4.66 to − 3.9)
North Africa and Middle East	11.85 (8.69–14.29)	18.32 (15.18–21.5)	54.62	0.57 (0.42–0.69)	0.38 (0.32–0.44)	− 1.59 (− 1.67 to − 1.52)
Oceania	0.33 (0.25–0.44)	0.55 (0.41–0.78)	67.15	0.78 (0.6–1)	0.59 (0.45–0.79)	− 1.06 (− 1.09 to − 1.03)
South Asia	44.97 (38.05–55.49)	80.44 (69.27–91.61)	78.87	0.64 (0.55–0.79)	0.56 (0.48–0.63)	− 0.58 (− 0.68 to − 0.48)
Southeast Asia	12.41 (10.18–17.69)	25.17 (19.85–29.4)	102.88	0.39 (0.33–0.57)	0.4 (0.31–0.46)	0.05 (− 0.08 to 0.17)
Southern Latin America	3.77 (3.47–4.09)	3 (2.69–3.35)	− 20.44	0.8 (0.73–0.87)	0.38 (0.34–0.43)	− 3.29 (− 3.71 to − 2.88)
Southern Sub-Saharan Africa	0.33 (0.25–0.42)	0.48 (0.36–0.6)	46.63	0.1 (0.07–0.12)	0.08 (0.06–0.09)	− 0.77 (− 1.37 to − 0.16)
Tropical Latin America	7.2 (6.88–7.55)	7.65 (7.26–8.15)	6.36	0.65 (0.62–0.68)	0.33 (0.31–0.35)	− 2.93 (− 3.34 to − 2.53)
Western Europe	99.5 (94.04–104.66)	61.62 (56.85–66.77)	− 38.07	1.78 (1.69–1.87)	0.69 (0.64–0.75)	− 4.12 (− 4.34 to − 3.89)
Western Sub-Saharan Africa	4.74 (3.75–5.92)	9.72 (7.87–12.33)	105.04	0.43 (0.34–0.54)	0.42 (0.33–0.52)	− 0.13 (− 0.16 to − 0.11)

*ASIR* age-standardized incidence rate, *CI* confidence interval, *EAPCs* estimated annual percentage changes, *UI* uncertainty interval

**Table 2:** The death cases and ASDR of CML in 1990 and 2017, and its temporal trends from 1990 to 2017

	Death cases no. *10^2^ (95% UI)		Change in absolute number (%)	ASDR per 100,000 No. (95% UI)		1990–2017 EAPCs No. (95% CI)
	1990	2017		1990	2017	
Overall	241.98 (226.32–261.08)	240.54 (222.33–260.72)	− 0.59	0.59 (0.56–0.63)	0.31 (0.28–0.33)	− 2.76 (− 2.89 to − 2.63)
Sex						
Male	125.44 (116.35–134.96)	129.15 (116.44–140.91)	2.96	0.69 (0.65–0.74)	0.36 (0.33–0.39)	− 2.66 (− 2.78 to − 2.53)
Female	116.54 (104.35–134.27)	111.39 (96.83–127.32)	− 4.42	0.53 (0.48–0.6)	0.26 (0.23–0.3)	− 2.9 (− 3.03 to − 2.76)
Socio-demographic index						
High SDI	115.87 (112.65–119.04)	70.66 (67.96–73.85)	− 39.02	0.92 (0.9–0.95)	0.31 (0.3–0.32)	− 4.58 (− 4.8 to − 4.36)
High-middle SDI	39.35 (35.19–43.45)	33.92 (31.8–37.07)	− 13.79	0.4 (0.36–0.44)	0.2 (0.18–0.21)	− 3.04 (− 3.27 to − 2.8)
Middle SDI	30.18 (27.14–35.29)	45.79 (39.63–49.07)	51.72	0.29 (0.26–0.33)	0.21 (0.18–0.23)	− 1.29 (− 1.39 to − 1.19)
Low-middle SDI	27.62 (24.17–34.96)	45.12 (39.06–52.46)	63.38	0.44 (0.39–0.56)	0.37 (0.32–0.43)	− 0.71 (− 0.82 to − 0.6)
Low SDI	28.54 (23.03–35.73)	44.67 (37.71–50.65)	56.53	0.77 (0.64–0.95)	0.6 (0.5–0.68)	− 1.03 (− 1.07 to − 0.98)
Region						
Andean Latin America	0.7 (0.59–0.81)	1.46 (1.19–1.64)	107.23	0.29 (0.25–0.33)	0.26 (0.21–0.29)	− 0.29 (− 0.42 to − 0.15)
Australasia	2.86 (2.74–3)	2.13 (1.89–2.43)	− 25.51	1.21 (1.15–1.26)	0.44 (0.39–0.5)	− 4.6 (− 4.88 to − 4.32)
Caribbean	2.23 (2.05–2.44)	2.45 (2.21–2.73)	9.81	0.81 (0.75–0.89)	0.48 (0.44–0.54)	− 2.04 (− 2.2 to − 1.87)
Central Asia	2.01 (1.72–2.29)	1.98 (1.75–2.2)	− 1.78	0.37 (0.32–0.43)	0.25 (0.22–0.27)	− 1.67 (− 1.86 to − 1.47)
Central Europe	9.76 (9.26–10.31)	6.42 (6.06–6.83)	− 34.21	0.67 (0.63–0.7)	0.31 (0.29–0.33)	− 3.07 (− 3.29 to − 2.86)
Central Latin America	5.57 (5.36–5.82)	8.55 (8.06–9.03)	53.44	0.57 (0.55–0.6)	0.36 (0.34–0.38)	− 2.02 (− 2.22 to − 1.83)
Central Sub-Saharan Africa	1.28 (0.87–1.62)	3.02 (2.21–3.75)	135.58	0.49 (0.34–0.61)	0.54 (0.39–0.67)	0.55 (0.45 to 0.65)
East Asia	14.93 (11.9–18.59)	11.79 (9.94–13.91)	− 21.05	0.14 (0.11–0.17)	0.06 (0.05–0.07)	− 3.56 (− 3.81 to − 3.3)
Eastern Europe	14.8 (13.44–16.76)	12.37 (11.71–13.09)	− 16.39	0.53 (0.48–0.6)	0.38 (0.36–0.4)	− 1.59 (− 1.95 to − 1.22)
Eastern Sub-Saharan Africa	9.74 (7.34–13.08)	13.98 (10.71–16.95)	43.43	1.06 (0.83–1.41)	0.77 (0.59–0.94)	− 1.4 (− 1.5 to − 1.3)
High-income Asia Pacific	10.33 (9.96–10.73)	6.01 (5.64–6.4)	− 41.8	0.52 (0.5–0.54)	0.14 (0.13–0.15)	− 5.12 (− 5.31 to − 4.94)
High-income North America	28.39 (27.68–29.2)	15.6 (14.97–16.43)	− 45.05	0.81 (0.79–0.83)	0.26 (0.25–0.27)	− 5.06 (− 5.49 to − 4.62)
North Africa and Middle East	9.66 (7.03–11.7)	12.32 (10.13–14.35)	27.53	0.51 (0.37–0.62)	0.28 (0.23–0.32)	− 2.26 (− 2.34 to − 2.19)
Oceania	0.26 (0.2–0.34)	0.41 (0.31–0.56)	56.59	0.66 (0.52–0.84)	0.47 (0.37–0.62)	− 1.27 (− 1.33 to − 1.21)
South Asia	38.65 (32.95–47.99)	66.6 (57.12–76.5)	72.32	0.6 (0.51–0.76)	0.49 (0.43–0.57)	− 0.8 (− 0.9 to − 0.71)
Southeast Asia	9.92 (8.24–14.64)	16.51 (13.01–19.91)	66.46	0.34 (0.29–0.5)	0.28 (0.22–0.33)	− 0.74 (− 0.88 to − 0.61)
Southern Latin America	3.21 (2.96–3.47)	2.29 (2.05–2.52)	− 28.84	0.69 (0.63–0.74)	0.28 (0.25–0.31)	− 3.87 (− 4.28 to − 3.46)
Southern Sub-Saharan Africa	0.27 (0.2–0.33)	0.37 (0.28–0.46)	40.37	0.08 (0.06–0.1)	0.06 (0.05–0.08)	− 0.87 (− 1.44 to − 0.3)
Tropical Latin America	5.8 (5.58–6.04)	5.97 (5.73–6.29)	2.84	0.56 (0.54–0.59)	0.26 (0.25–0.28)	− 3.23 (− 3.59 to − 2.87)
Western Europe	67.54 (64.82–70.15)	42.45 (40.25–44.87)	− 37.16	1.17 (1.13–1.21)	0.44 (0.41–0.46)	− 4.16 (− 4.4 to − 3.92)
Western Sub-Saharan Africa	4.05 (3.17–5.07)	7.87 (6.28–9.9)	94.46	0.4 (0.31–0.5)	0.37 (0.3–0.47)	− 0.21 (− 0.23 to − 0.19)

ASDR age-standardized death rate, CI confidence interval, EAPCs estimated annual percentage changes, UI uncertainty interval

**Table 3:** The DALYs and age-standardized DALYs Rate of CML in 1990 and 2017, and its temporal trends from 1990 to 2017

	DALYs No. *10^3^ (95% UI)		Change in absolute number (%)	Age-standardized DALY rate per 100,000 no. (95% UI)		1990–2017 EAPCs No. (95% CI)
	1990	2017		1990	2017	
Overall	734.92 (666.26–806.31)	654.98 (594.73–712.95)	− 10.88	15.96 (14.6–17.44)	8.17 (7.42–8.88)	− 2.84 (− 2.98 to − 2.69)
Sex						
Male	382.94 (349.15–415.35)	347.24 (308.67–383.12)	− 9.32	17.48 (16.08–18.92)	9 (8.01–9.9)	− 2.79 (− 2.93 to − 2.65)
Female	351.99 (299.2–414.08)	307.74 (258.51–357.55)	− 12.57	14.78 (12.72–17.36)	7.48 (6.27–8.68)	− 2.89 (− 3.04 to − 2.75)
Socio-demographic index						
High SDI	286.21 (278.31–294.78)	125.91 (121.16–131.32)	− 56.01	24.23 (23.53–24.96)	6.81 (6.57–7.08)	− 5.34 (− 5.6 to − 5.08)
High-middle SDI	129.2 (112.36–144.95)	91.51 (85–100.26)	− 29.17	12.17 (10.67–13.57)	5.26 (4.9–5.76)	− 3.58 (− 3.84 to − 3.32)
Middle SDI	110.8 (96.98–129.21)	137.8 (120.1–148.24)	24.37	8.68 (7.68–10.09)	6.03 (5.25–6.47)	− 1.6 (− 1.73 to − 1.48)
Low-middle SDI	100.72 (86–128.82)	146.73 (127.78–171.91)	45.68	13.25 (11.49–16.8)	10.33 (8.95–12.07)	− 1 (− 1.14 to − 0.87)
Low SDI	106.41 (82.75–137.73)	151.91 (126.85–172.16)	42.76	23.14 (18.45–29.19)	16.71 (14.03–18.84)	− 1.35 (− 1.42 to − 1.28)
Region						
Andean Latin America	2.58 (2.16–2.98)	4.49 (3.61–5.14)	73.96	9.01 (7.53–10.28)	7.68 (6.21–8.76)	− 0.45 (− 0.59 to − 0.32)
Australasia	6.8 (6.49–7.13)	4.02 (3.54–4.58)	− 40.85	29.19 (27.86–30.62)	9.87 (8.64–11.2)	− 4.84 (− 5.13 to − 4.54)
Caribbean	6.72 (6.12–7.52)	6.66 (5.9–7.62)	− 0.77	22.56 (20.54–25.15)	13.28 (11.77–15.18)	− 2.11 (− 2.28 to − 1.93)
Central Asia	7.39 (6.31–8.47)	6.69 (5.93–7.44)	− 9.46	12.76 (10.93–14.6)	7.62 (6.77–8.45)	− 2.08 (− 2.32 to − 1.84)
Central Europe	27.55 (26.09–29.01)	13.52 (12.8–14.32)	− 50.92	19.12 (18.14–20.16)	7.53 (7.14–7.98)	− 3.76 (− 3.97 to − 3.55)
Central Latin America	19.46 (18.71–20.35)	24.79 (23.35–26.32)	27.39	16.88 (16.22–17.64)	9.98 (9.41–10.58)	− 2.34 (− 2.55 to − 2.13)
Central Sub-Saharan Africa	4.86 (3.14–6.32)	11.01 (7.99–13.7)	126.38	14.47 (9.83–18.41)	14.97 (10.91–18.55)	0.26 (0.16 to 0.36)
East Asia	62.56 (46.66–79.09)	36.19 (30.76–43.35)	− 42.15	5.02 (3.85–6.27)	1.9 (1.63–2.27)	− 4.37 (− 4.73 to − 4.02)
Eastern Europe	44.34 (40.1–50.69)	32.48 (30.66–34.41)	− 26.74	16.35 (14.87–18.62)	10.85 (10.26–11.55)	− 2.04 (− 2.47 to − 1.61)
Eastern Sub-Saharan Africa	37.33 (26.7–51.97)	51.15 (39.01–62.33)	37.04	32.79 (24.4–43.98)	21.72 (16.64–26.33)	− 1.82 (− 1.95 to − 1.69)
High-income Asia Pacific	34.19 (32.85–35.83)	11.61 (10.84–12.33)	− 66.04	17.16 (16.47–18.02)	3.85 (3.59–4.12)	− 5.94 (− 6.17 to − 5.72)
High-income North America	73.89 (71.98–76.1)	31.36 (29.89–33.21)	− 57.56	22.53 (21.93–23.2)	6.14 (5.82–6.5)	− 5.78 (− 6.24 to − 5.33)
North Africa and Middle East	33.74 (24.28–41.55)	39.69 (32.3–47.11)	17.63	14.58 (10.63–17.84)	7.66 (6.27–9)	− 2.49 (− 2.56 to − 2.41)
Oceania	1.06 (0.79–1.44)	1.65 (1.2–2.34)	55.08	23.03 (17.52–30.17)	16.36 (12.26–22.41)	− 1.29 (− 1.36 to − 1.23)
South Asia	140.17 (116.31–172.87)	207.79 (180.43–239.39)	48.24	17.35 (14.7–21.55)	13.25 (11.44–15.19)	− 1.08 (− 1.19 to − 0.96)
Southeast Asia	36.28 (28.98–52.09)	52.2 (41.39–62.2)	43.89	10.3 (8.45–15)	7.87 (6.24–9.39)	− 1.02 (− 1.16 to − 0.87)
Southern Latin America	9.22 (8.5–9.93)	5.36 (4.81–5.91)	− 41.85	19.28 (17.79–20.78)	7 (6.27–7.73)	− 4.32 (− 4.73 to − 3.91)
Southern Sub-Saharan Africa	0.98 (0.73–1.19)	1.26 (0.93–1.57)	29.18	2.63 (1.96–3.24)	1.88 (1.39–2.3)	− 1.15 (− 1.8 to − 0.49)
Tropical Latin America	20.5 (19.63–21.45)	15.79 (15.04–16.59)	− 22.97	16.99 (16.31–17.74)	6.64 (6.33–6.97)	− 3.92 (− 4.32 to − 3.52)
Western Europe	150.45 (144.73–156.46)	68.46 (64.8–72.73)	− 54.49	28.93 (27.85–30.07)	8.84 (8.39–9.38)	− 4.97 (− 5.25 to − 4.69)
Western Sub-Saharan Africa	14.87 (11.7–18.64)	28.79 (23.26–37.07)	93.7	12.23 (9.58–15.37)	10.82 (8.63–13.73)	− 0.51 (− 0.55 to − 0.47)

*DALYs* disability-adjusted life years, *CI* confidence interval, *EAPCs* estimated annual percentage changes, *UI* uncertainty interval

### Statistical analysis

We used annual incidence cases, death cases, DALYs, and its corresponding age-standardized rate (ASR) to describe the CML burden. Age-standardized incidence rate (ASIR), age-standardized death rate (ASDR), and age-standardized DALYs rate could ensure comparability among populations with different age structures and population sizes. Estimated annual percentage changes (EAPCs) were calculated based on ASR and employed to quantify the trend of ASR. Computational formulas of EAPCs were *y* = *α* + *βx* + *ε* and *EAPCs* = *100* × *(exp(β) − 1)*, where *y, x* referred to *ln(ASR)* and calendar year, respectively. ASR showed an upward trend if EAPCs and its 95% confidence interval (CI) were positive. Inversely, ASR showed a downward trend if EAPCs and its 95% CI were negative. Besides, we used Pearson's correlation coefficient to explore further the relationship between ASR and SDI. All statistical analyses and figures were performed using R software (version 3.6.3).

## Results

### Incidence burden of CML

**Fig. 1:**
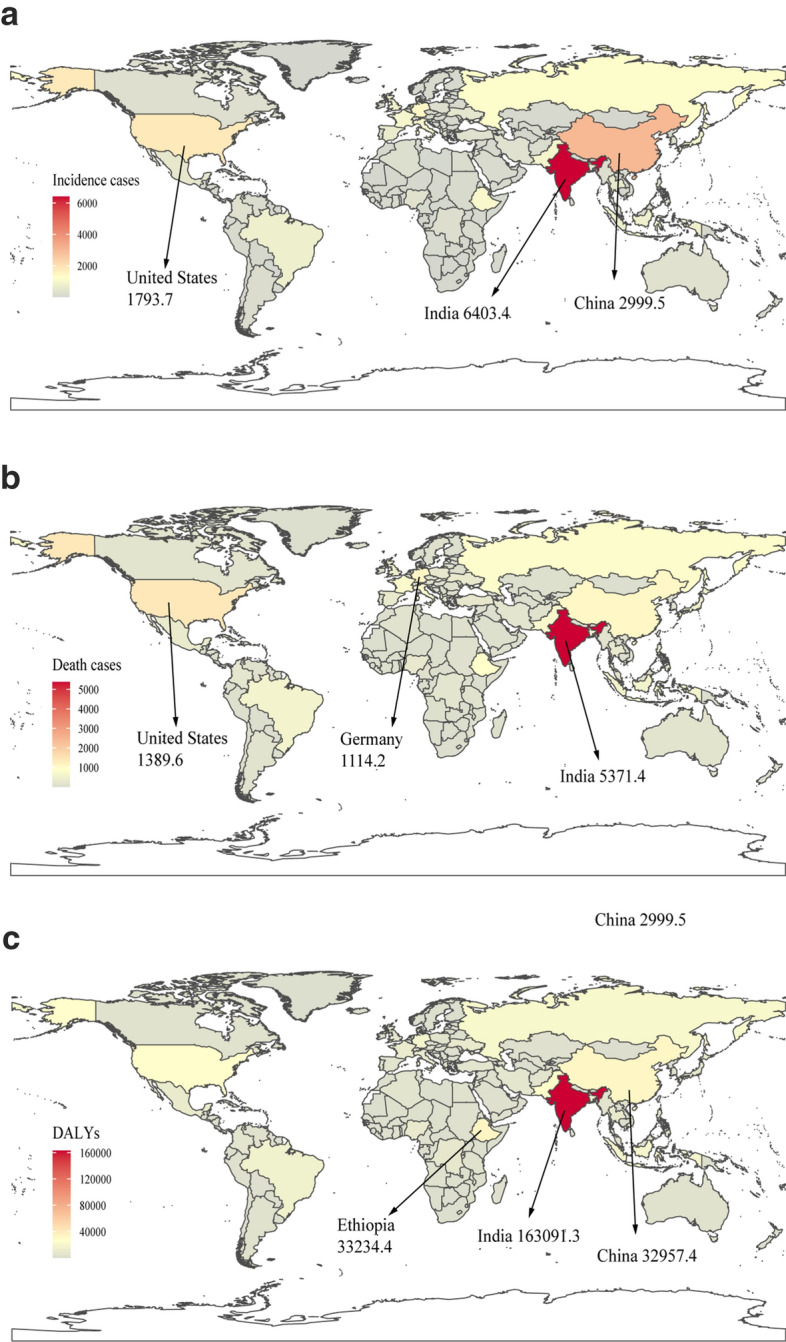
The global disease burden of CML in 195 countries or territories in 2017: **a** The incidence cases of 195 countries or territories in 2017; **b** the deaths of 195 countries or territories in 2017; **c** the DALYs of 195 countries or territories in 2017. CML, chronic myeloid leukemia; DALYs, disability-adjusted life years

**Fig. 2:**
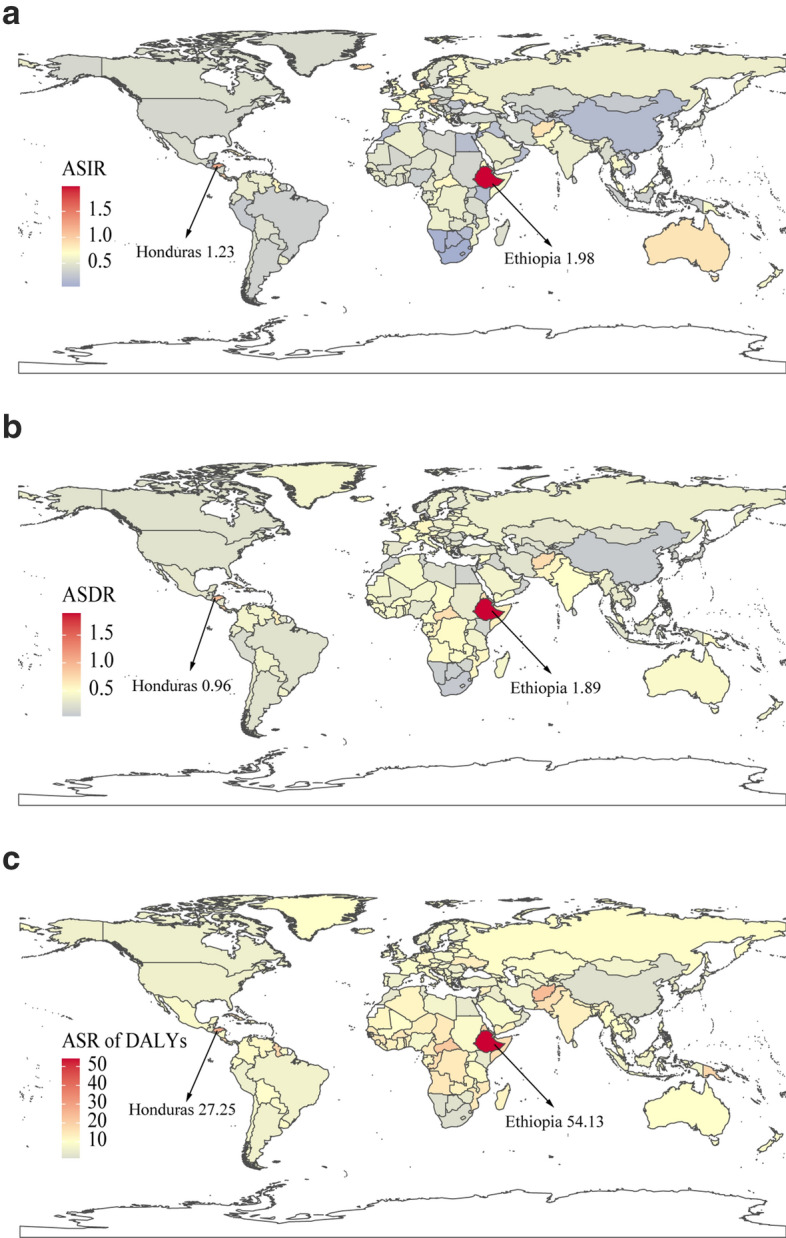
The age-standardized rates of CML in 195 countries or territories in 2017: **a** The ASIR of 195 countries or territories in 2017; **b** the ASDR of 195 countries or territories in 2017; **c** the age-standardized DALYs rate of 195 countries or territories in 2017. CML, chronic myeloid leukemia; ASIR, age-standardized incidence rate; ASDR, age-standardized death rate; DALYs, disability-adjusted life year

Globally, incidence cases of CML increased slightly from 31,752 in 1990 to 34,179 in 2017, but the ASIR decreased significantly from 0.75/100,000 persons in 1990 to 0.43/100,000 persons in 2017. At the SDI quintiles level, incidence cases were decreasing in the high SDI quintiles, but increasing in the other four SDI quintiles from 1990 to 2017. Additionally, in 1990, high SDI quintiles had the highest ASIR at 1.34/100,000 persons. Alarmingly, in 2017, low SDI quintiles had the highest ASIR at 0.65/100,000 persons, which was higher than the ASIR in high SDI quintiles at 0.53/100,000 persons. The ASIR of the five SDI quintiles remained a downward trend, and high SDI quintiles had the most considerable decline with EAPCs at − 4 (95% CI − 4.23 to − 3.27). In terms of geographical regions, Western Europe with incidence cases at 6,162 in 2017 and South Asia with incidence cases at 8,044 in 2107 stayed the top two most incidence cases in the world. From 1990 to 2017, the ASIR showed a decreasing trend in most geographical regions except in Central Sub-Saharan Africa and Andean Latin America (Table 1). Regarding observation of countries and territories, in 2017, India, China, and the United States had the most incidence cases at 6403.4, 2999.5, and 1793.7, respectively (Fig. 1a, Additional file 1: Table S1). Ethiopia had the highest ASIR at 1.98/100,000 persons in 2017 (Fig. 2a, Additional file 1: Table S2). Jamaica and Germany had the most increase and decline in ASIR, respectively (Additional file 1: Table S3, Figure S1a).

### Deaths and DALYs burden of CML

The death cases stayed stable globally, with 24,198 cases in 2017, and DALYs decreased slightly to 654,980 years in 2017. From 1990 to 2107, the ASDR and age-standardized DALYs rate of CML decreased significantly. At the SDI quintiles level, death cases and DALYs were decreasing in the high SDI and high-middle SDI quintiles, but increasing in the other three SDI quintiles from 1990 to 2017. Additionally, high SDI quintiles had the highest ASDR and age-standardized DALYs rate in 1990, but in 2017, the low SDI quintiles had the highest values. The high SDI quintiles had a considerable decrease in ASDR with EAPCs at − 4.58 and age-standardized DALYs rate with EAPCs at − 5.34. In terms of geographical regions, Western Europe and South Asia were the top two regions with most death cases and DALYs in 2017. The ASDR and age-standardized DALYs rate showed a decreasing trend in all geographical regions except in Central Sub-Saharan Africa (Tables 2, 3). Regarding observation of countries and territories, in 2017, India had the most death cases and DALYs (Fig. 1b, c, Additional file 1: Table S1). Ethiopia had the highest ASDR at 1.89/100,000 persons, and age-standardized DALYs at 54.13/100,000 persons in 2017 (Fig. 2b, c, Additional file 1: Table S2). Jamaica and Japan had the most increase and decline in ASIR and age-standardized DALYs (Additional file 1: Table S3, Figure S1b and c).

### Sex and age distribution of incidence, deaths and DALYs

**Fig. 3:**
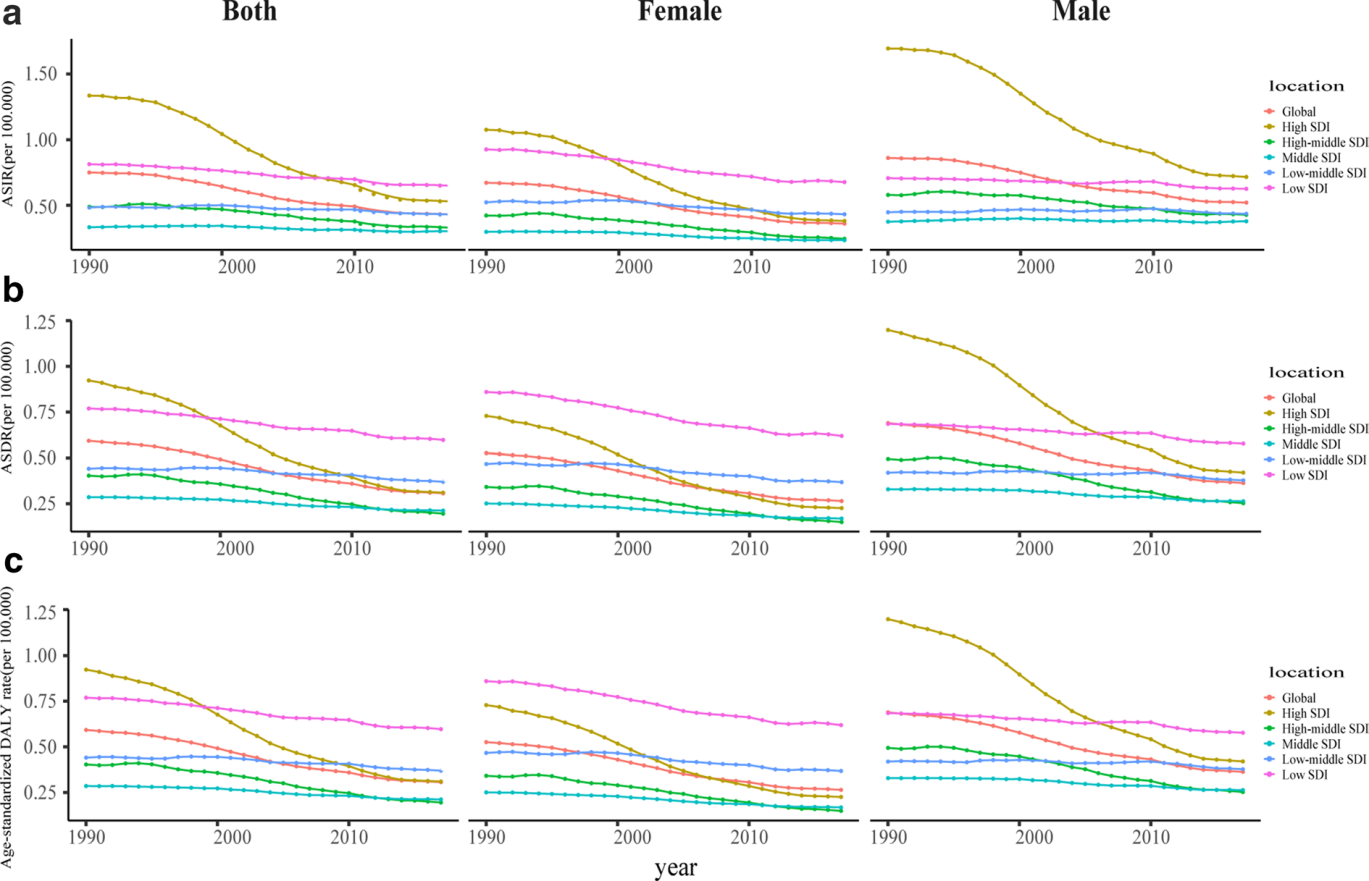
The change trends of age-standardized rates of CML among different SDI quintiles and sex: **a** the ASIR from 1990 to 2017; **b** The ASDR from 1990 to 2017; **c** The age-standardized DALYs rate from 1990 to 2017. CML, chronic myeloid leukemia; ASIR, age-standardized incidence rate; ASDR, age-standardized death rate; DALYs, disability-adjusted life year; SDI, socio-demographic index

**Fig. 4:**
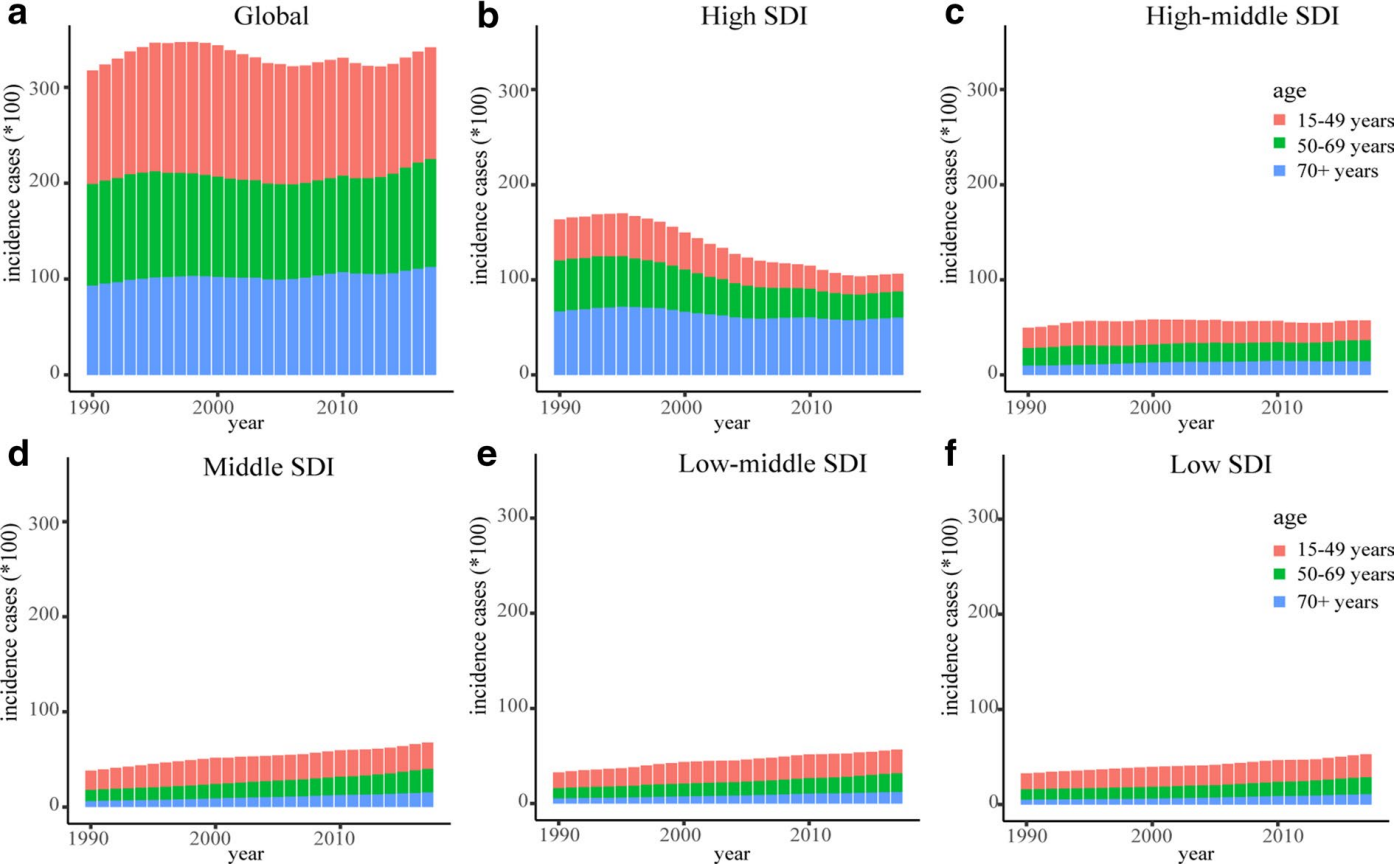
The incidence cases of CML in three age groups from 1990 to 2017: **a** The incidence cases in the globe; **b** The incidence cases in the high SDI quintiles; **c** The incidence cases in the high-middle SDI quintiles; **d** The incidence cases in the middle SDI quintiles; **e** The incidence cases in the low-middle SDI quintiles; **f** The incidence cases in the low SDI quintiles. The three age groups included 15–49 years, 50–69 years, and 70 + years. SDI, socio-demographic index

Globally, the incidence and death cases of CML increased slightly in males while decreased in females. The ASR in males was higher than in females. At the SDI quintiles level, for males, high SDI quintiles always had the highest ASIR between 1990 to 2017; high SDI quintiles had the highest ASDR and age-standardized DALYs rate in 1990, but in 2017, low SDI quintiles had the highest values. For females, high SDI quintiles had the highest ASIR in 1990, but in 2017, low SDI quintiles had the highest values; low SDI quintiles always had the highest ASDR and age-standardized DALYs rate between 1990 to 2017 (Fig. 3). Additionally, the incidence, death, and DALY rate increased with age (Additional file 1: Figure S2). The higher the SDI, the lower proportion of the young cases of incidence, deaths, and DALYs, but the higher proportion of the elderly cases (Fig. 4, Additional file 1: Figures S3, 4). The proportion of elderly incidence and death cases increased with years, and death cases were mainly concentrated in the age group over 70 years (Additional file 1: Figure S5).

### The influential factors for EAPCs

**Fig. 5:**
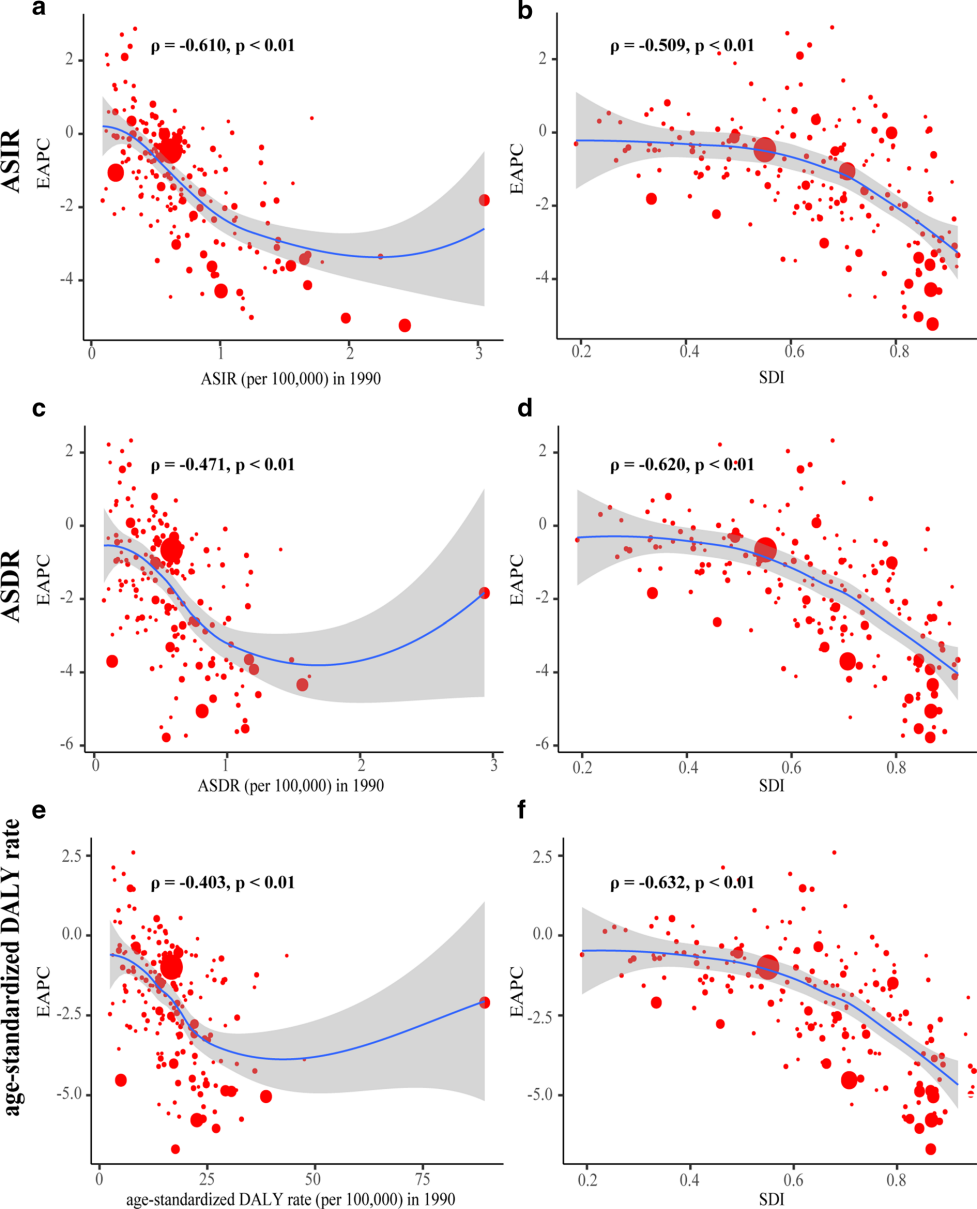
The correlation between EAPCs and age-standardized rate in 1990, and SDI in 2017: The correlation between EAPCs and ASIR in 1990 (**a**), and SDI in 2017 (**b**); The correlation between EAPCs and ASDR in 1990 (**c**), and SDI in 2017 (**d**); The correlation between EAPCs and age-standardized DALYs rate in 1990 (**e**), and SDI in 2017 (**f**). The circles represent 195 countries or territories and the size of circle represents the number of CML patients. ρ, Pearson’s correlation coefficient; CML, chronic myeloid leukemia; ASIR, age-standardized incidence rate; ASDR, age-standardized death rate; DALYs, disability-adjusted life year; SDI, socio-demographic index; EAPCs estimated annual percentage changes

We evaluated the correlation coefficient between the EAPCs and ASR in 1990, and the SDI in 2017. We found that ASIR (ρ = − 0.610, p < 0.01), ASDR (ρ = − 0.471, p < 0.01) and age-standardized DALY rate (ρ = − 0.403, p < 0.01) in 1990 was negatively correlated with its corresponding EAPCs. Meanwhile, correlations between SDI and EAPCs of incidence (ρ = − 0.509, p < 0.01), deaths (ρ = − 0.620, p < 0.01) and DALYs (ρ = -0.632, p < 0.01) were significant negative. These findings indicated the ASR of countries with larger disease reservoir baseline in 1990 or higher SDI in 2017 might show a faster descending trend (Fig. 5).

### CML burden attributable to risk factors

**Fig. 6:**
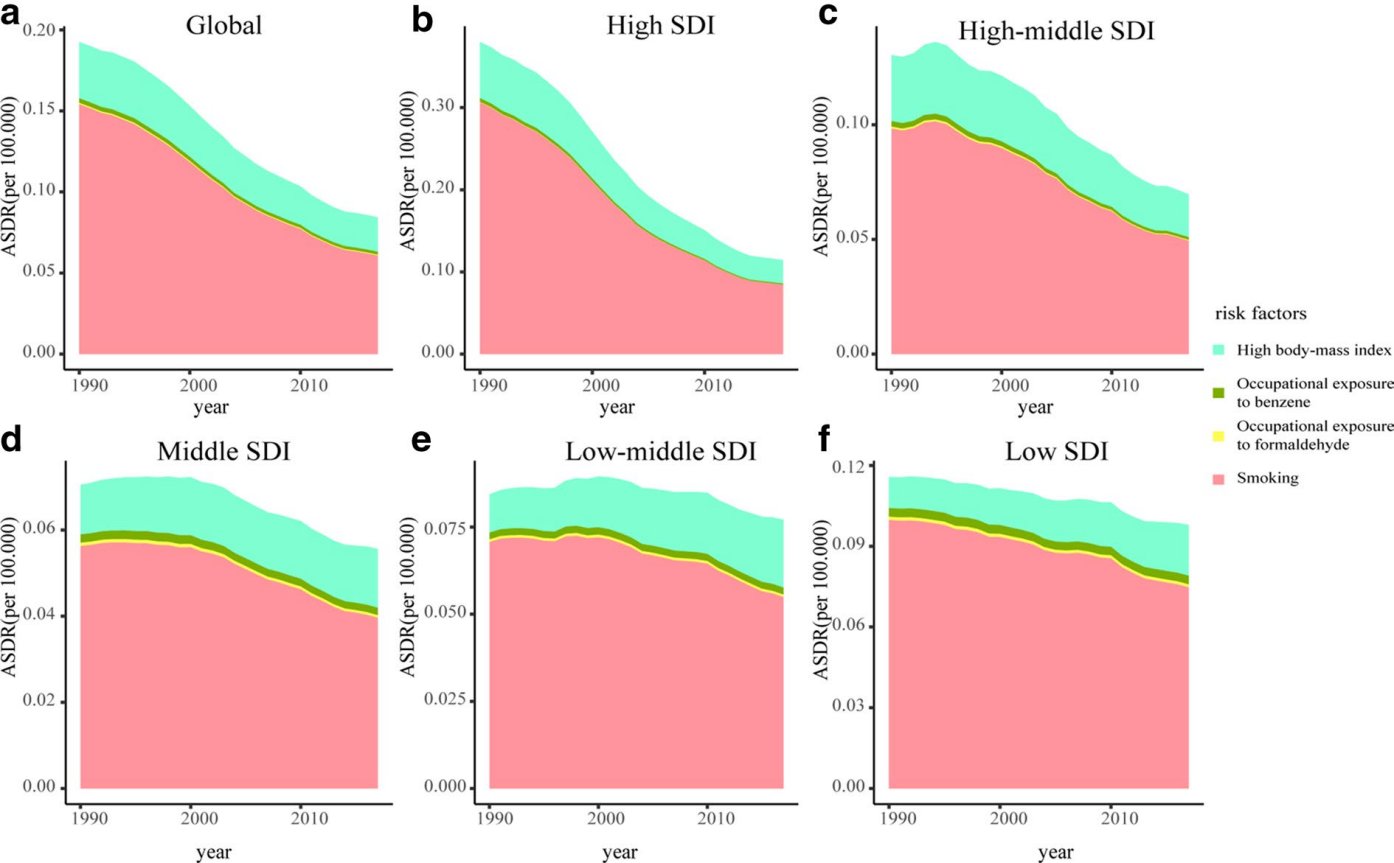
The age-standardized rates of CML deaths attributed to risk factors from 1990 to 2017 in Global (**a**), High SDI (**b**), High-middle SDI (**c**), Middle SDI (**d**), Low-middle SDI (**e**), Low SDI (**f**). CML, chronic myeloid leukemia; ASDR, age-standardized death rate; SDI, socio-demographic index

From 1990 and 2017, smoking, high body mass index, and occupational exposure to benzene or formaldehyde were the potential risk factors related to CML burden in the GBD study, of which smoking was the most significant contributor. At the SDI quintiles level, CML deaths and DALYs attributed to smoking decreased quickly from 1990 to 2017 in high SDI quintiles and declined slightly in the other four SDI quintiles. Besides, the higher the SDI, the higher the ASR of CML deaths and DALYs attributed to risk factors (Fig. 6, Additional file 1: Figure S6). Globally, the contribution ratio of smoking descended from 1990 to 2017, accounting for 19.8% of CML deaths and 15.8% of DALYs in 2017. Meanwhile, the contribution ratio of high body mass index increased slightly and led to 6.94% of deaths, and 6.52% of DALYs in 2107. The percent of CML deaths and DALYs attributed to occupational exposure to benzene or formaldehyde were less than 1% (Additional file 1: Figures S7, 8).

## Discussion

In this study, we assessed trends in the disease burden of CML based on GBD study, providing valuable epidemiologic information for health promotion and disease prevention. Though the ASIR, ASDR, and age-standardized DALYs rate generally declined, the worldwide disease burden of CML appeared to be stable due to population growth in developing countries and population aging in developed countries [17]. From 1990 to 2017, incidence cases decreased by 34.9% in high SDI quintiles but increased by over 60% in low SDI, low-middle SDI, and middle SDI quintiles. Similarly, Andean Latin America, Central Sub-Saharan Africa, South Asia, and Western Sub-Saharan Africa with lower SDI noticed the fastest growth in the incidence cases, death cases, and DALYs. Besides, India had the most incidence cases, death cases, and DALYs of CML in the globe, leading to the highest disease burden, which primarily due to a vast population base. Developed countries generally obtained remarkable achievements in reducing the disease burden due to CML [9, 18]. TKI-based therapy was the foremost reason for the profound improvement of survival in CML patients [7]. Currently, developing and low-income countries gradually introduced novel drugs like imatinib [19]. However, CML patients in these countries still had difficulties accessing TKI-based therapy or may receive delayed therapy, thanks to the limited availability of novel drugs [20]. Additionally, the high cost of medication and monitoring was another challenge for patients in low-income countries [21, 22]. Generic drug application and international patient assistance programs can reduce the economic burden of CML patients in countries with limited resources.

It is worth mentioning that treatment-free remission (TFR) is a new goal for many CML patients, as TKI-based therapy requires lifelong treatment and significantly aggravates economic burden [23, 24]. A prospective trial in France first revealed some CML patients with a stable deep molecular response could cease their TKI therapy safely without relapsing, known as TFR [25]. Since then, multiple clinical trials were conducted to explore the criteria of cessation attempts and triggers for resuming TKI therapy [26, 27]. In most studies, the molecular recurrence rate of TKI cessation was about 50%. Meanwhile, at least three years of TKI therapy and one-year deep molecular response were the criteria of cessation attempts, and loss of major molecular response met the triggers for resuming TKI therapy. The financial burden of health agencies became more massive based on lifelong TKI treatment with increasing cases of CML patients around the world. Besides, lifelong therapy is also associated with adverse events related to therapy, resulting in lower life quality and a growing disease burden of CML patients [28]. Women with childbearing potential must use effective contraception during TKI therapy and avoid breastfeeding due to evidence of teratogenicity. TFR successfully achieved in some CML patients can help to reduce disease burden. However, it should be emphasized that frequent monitoring and planned follow-up were essential requirements for discontinuing TKI therapy. Countries with inadequate resources might face challenges in ensuring necessary monitoring and effective drugs; thus, improving survival rate rather than TFR was the primary goal of CML patients in these countries [29]. Developed countries could conduct more research to establish the TFR criterion, which can be a critical initiative in reducing CML's disease burden.

The epidemiologic trend of CML differed in age and sex, which was significant for policymakers [30]. Aging, related to a decrease of hematopoietic stem cell function, was an essential factor associated with leukemogenesis [31, 32]. Moreover, CML survival was age-related, and age was an essential factor for treatment options [33, 34]. Thus, higher SDI quintiles with population aging showed a more substantial proportion of older patients over 70 years old. Males had a higher risk to CML than females, and the male to female ratio fluctuated between 1.2 and 1.7 [35, 36]. In general, females had a better survival rate of CML than males, which was in line with other malignant diseases [35, 37]. Better general longevity and hormonal status of females and environmental and genetic factors might impact the age distribution of CML patients [38, 39]. However, in the low SDI quintiles, females had a higher age-standardized rate of deaths and DALYs than males in 2017. Previous studies also found that women in low-income countries tended to have higher mortality due to inadequate opportunities for screening and early treatment [40, 41]. Besides, the trend changes in smoking, obesity and physical inactivity in these countries may also contribute to the increasing disease burden of females. Prioritizing the reduction of known disease risk factors in low-income countries may be an effective intervention to reduce the health burden. Given the scarcity of resources, the support and commitment of the international community are also imperative.

Smoking was always the leading potential factor contributing to CML deaths and DALYs, based on data in the GBD study, though its contribution ratio decreased. Attributable deaths and DALYs due to smoking descended most quickly in high SDI quintiles and declined slightly in the other four SDI quintiles. These findings emphasized the urgent need to strengthen smoking control to reduce the CML burden. A previous study revealed that smoking might be an adverse prognostic factor, shortening survival time and contributing to CML's progression [42]. The smoking prevalence declined in most countries during the research, especially in high-income countries [43]. The ratio of former smokers to current smokers in middle age was a reliable indicator of smoking cessation; the ratio was about one in developed countries, implying partial successful quitting [44]. Worryingly, low-income and middle-income countries, with approximately 80% of all smokers living in, had far fewer former smokers than current smokers [44]. Actually, the smoking population in many developing countries has boomed as their affordability for cigarettes since 1990. Reviews of comprehensive cessation programs highlight higher prices are particularly useful [45]. Besides, non-price interventions include warning labels, media campaigns, and assistance with smoking cessation could also increase quit rates [46]. High body mass index was the second attributable risk factor after smoking; its contribution ratio declined slightly in high SDI and high-middle SDI quintiles but increased in the other three SDI quintiles. Prospective studies also indicated a positive correlation between obesity and leukemia mortality [47, 48]. In the past decade, the obesity epidemic has leveled off in developed countries; by contrast, an increasing trend was still evident in developing countries [49, 50]. The obesity burden seemed to shift to the poor [51]. Since the twenty-first century, urbanization was a critical driver in obesity increasing in developing countries; production of cheap vegetable oils, allowing increasing consumption of energy- and fat-rich diet at low income, and sedentary lifestyles also forced the obesity epidemic [50, 51]. Providing consultation and screening in the health care system, and reducing the marketing of sugary beverages and nonessential high‐calorie foods could help reduce attributable CML deaths.

A recent study also assessed the global burden of acute myeloid leukemia (AML) based on the GBD study. The significant difference in the global burden between CML and AML was a downward trend in CML and an upward trend in AML [52]. In 2017, high SDI quintiles tended to have a lower incidence and death rate of CML than low SDI quintiles, while AML was the opposite. CML's disease risk was similar to AML in terms of age and sex distribution, which was increased by age and a higher chance in males. As for attributable risks of CML and AML, the trend was consistent, and smoking was the leading attributable risk factor, followed by high body mass index. TKI-based therapy has drastically changed the landscape of CML survival, while novel effective therapies are incredibly urgent for AML patients [52]. Policymakers could pay more attention to reducing the CML burden in lower SDI countries. Besides, establishing the TFR criterion could be a critical initiative in reducing CML's disease burden.

Some unavoidable limitations did exist in this study. The data from the GBD study could fill the gap since actual data of CML burden was unavailable. The accuracy of our study depended on the quality of data in the GBD study. Additionally, differences in data acquiring and data source quality in the GBD study could be inevitable. Minor fluctuation in age-standardized rates may be associated with adjustments of disease screening strategies instead of real changes. GBD study lacked data about race, which can help better explain the distribution of CML.

## Conclusion

Globally, the disease burden of CML has been decreasing from 1990 to 2017. Expressly, the incidence, deaths, and DALYs rate declined sharply in high SDI and high-middle quintiles and a minor decline in the other three SDI quintiles. Additionally, the incident cases, death cases, and DALYs of CML showed an upward trend in middle SDI, low-middle SDI, and low SDI quintiles between 1990 to 2017 due to population growth. In recent years, higher SDI countries achieved remarkable achievements in diminishing CML burden. Lower SDI countries should pay more attention to reducing the disease burden of CML. Consequently, strategies about early detection of CML, the introduction of novel drugs, and against attributable factors such as smoking and high body mass index should be implemented to reduce CML burden, especially in lower SDI quintiles.

## Supplementary information

**Additional file 1: Table S1.**Top 10 countries or territories with most incidence, death or DALYs cases in 2017.** Table S2**. Top 10 countries or territories with highest age-standardized rate of incidence, deaths, or DALYs in 2017.** Table S3 .**Top 5 countries or territories with the most increase and decrease in age-standardized rate of incidence, deaths, or DALYs from 1990 to 2017.** Figure S1.** The global EAPCs of CML in 195 countries or territories in 2017: a The EAPCs of incidence in 2017; b the EAPCs of deaths in 2017; c the EAPCs of DALYs in 2017. CML, chronic myeloid leukemia; DALYs, disability-adjusted life years; EAPCs estimated annual percentage changes.** Figure S2.** The incidence, death, and DALY rates of CML in different age groups: a The incidence rate in 1990; b The incidence rate in 2017; c The death rate in 1990; d The death rate in 2017; e The DALYs rate in 1990; f The DALYs rate in 2017. CML, chronic myeloid leukemia; DALYs, disability-adjusted life years.** Figure S3 .**The death cases of CML in three age groups from 1990 to 2017: a The death cases in the globe; b The death cases in the high SDI regions; c The death cases in the high-middle SDI regions; d The death cases in the middle SDI regions; e The death cases in the low-middle SDI regions; f The death cases in the low SDI regions. The three age groups included 15–49 years, 50–69 years, and 70+ years. SDI, socio-demographic index.** Figure S4 .**The DALYs of CML in three age groups from 1990 to 2017: a The DALYs in the globe; b The DALYs in the high SDI regions; c The DALYs in the high-middle SDI regions; d The DALYs in the middle SDI regions; e The DALYs in the low-middle SDI regions; f The DALYs in the low SDI regions. The three age groups included 15–49 years, 50–69 years, and 70+ years. SDI, socio-demographic index; DALYs, disability-adjusted life years.** Figure S5.** The proportion of different ages and sex in CML incidence cases (a) and death cases (b), and DALYs (c) from 1990 to 2017. DALYs, disability-adjusted life years.** Figure S6.** The age-standardized rates of CML DALYs attributed to risk factors from 1990 to 2017 in Global (a), High SDI (b), High-middle SDI (c), Middle SDI (d), Low-middle SDI (e), Low SDI (f). CML, chronic myeloid leukemia; SDI, socio-demographic index. DALYs, disability-adjusted life years.** Figure S7.** The percent of CML deaths attributed to risk factors from 1990 to 2017 in Global (a), High SDI (b), High-middle SDI (c), Middle SDI (d), Low-middle SDI (e), Low SDI (f). CML, chronic myeloid leukemia; SDI, socio-demographic index.** Figure S8 .**The percent of CML DALYs attributed to risk factors from 1990 to 2017 in Global (a), High SDI (b), High-middle SDI (c), Middle SDI (d), Low-middle SDI (e), Low SDI (f). CML, chronic myeloid leukemia; SDI, socio-demographic index. DALYs, disability-adjusted life years

## Data Availability

The datasets generated during and/or analyzed during the current study are available from the Global Health Data Exchange query tool (https://ghdx.healthdata.org/gbd-results-tool).

## Abbreviations

CML: Chronic myeloid leukemia
ASR: Age-standardized rate
ASIR: Age-standardized incidence rate
ASDR: Age-standardized death rate
EAPCs: Estimated annual percentage change
DALYs: Disability-adjusted life-years
GBD: Global burden of disease
SDI: Social-demographic index
UI: Uncertainty interval
CI: Confidence interval
TKI: Tyrosine kinase inhibitor
TFR: Treatment-free remission
