# Supplementary material for: Trends in disease burden of chronic myeloid leukemia at the global, regional, and national levels: a population-based epidemiologic study
Source: Exp Hematol Oncol. 2020 Nov 3;9:29. doi: 10.1186/s40164-020-00185-z (PMC7607878; doi:10.1186/s40164-020-00185-z)
Supplement: Supplementary file 1 — Additional file 1: Table S1.Top 10 countries or territories with most incidence, death or DALYs cases in 2017. Table S2. Top 10 countries or territories with highest age-standardized rate of incidence, deaths, or DALYs in 2017. Table S3 .Top 5 countries or territories with the most increase and decrease in age-standardized rate of incidence, deaths, or DALYs from 1990 to 2017. Figure S1. The global EAPCs of CML in 195 countries or territories in 2017: a The EAPCs of incidence in 2017; b the EAPCs of deaths in 2017; c the EAPCs of DALYs in 2017. CML, chronic myeloid leukemia; DALYs, disability-adjusted life years; EAPCs estimated annual percentage changes. Figure S2. The incidence, death, and DALY rates of CML in different age groups: a The incidence rate in 1990; b The incidence rate in 2017; c The death rate in 1990; d The death rate in 2017; e The DALYs rate in 1990; f The DALYs rate in 2017. CML, chronic myeloid leukemia; DALYs, disability-adjusted life years. Figure S3 .The death cases of CML in three age groups from 1990 to 2017: a The death cases in the globe; b The death cases in the high SDI regions; c The death cases in the high-middle SDI regions; d The death cases in the middle SDI regions; e The death cases in the low-middle SDI regions; f The death cases in the low SDI regions. The three age groups included 15–49 years, 50–69 years, and 70+ years. SDI, socio-demographic index. Figure S4 .The DALYs of CML in three age groups from 1990 to 2017: a The DALYs in the globe; b The DALYs in the high SDI regions; c The DALYs in the high-middle SDI regions; d The DALYs in the middle SDI regions; e The DALYs in the low-middle SDI regions; f The DALYs in the low SDI regions. The three age groups included 15–49 years, 50–69 years, and 70+ years. SDI, socio-demographic index; DALYs, disability-adjusted life years. Figure S5. The proportion of different ages and sex in CML incidence cases (a) and death cases (b), and DALYs (c) from 1990 to 2017. DALYs, disability-adju [file 40164_2020_185_MOESM1_ESM.doc]

**Supplementary Tables**

**Table S1** Top 10 countries or territories with most incidence, death or DALYs cases in 2017.

**Table S2** Top 10 countries or territories with highest age-standardized rate of incidence, deaths, or DALYs in 2017.

**Table S3** Top 5 countries or territories with the most increase and decrease in age-standardized rate of incidence, deaths, or DALYs from 1990 to 2017.

**Supplementary Figures**

**Figure S1** The global EAPCs of CML in 195 countries or territories in 2017: **a** The EAPCs of incidence in 2017; **b** the EAPCs of deaths in 2017; **c** the EAPCs of DALYs in 2017. CML, chronic myeloid leukemia; DALYs, disability-adjusted life years; EAPCs estimated annual percentage changes

**Figure S2** The incidence, death, and DALY rates of CML in different age groups: **a** The incidence rate in 1990; **b** The incidence rate in 2017; **c** The death rate in 1990; **d** The death rate in 2017; **e** The DALYs rate in 1990; **f** The DALYs rate in 2017. CML, chronic myeloid leukemia; DALYs, disability-adjusted life years

**Figure S3** The death cases of CML in three age groups from 1990 to 2017: **a** The death cases in the globe; **b** The death cases in the high SDI regions; **c** The death cases in the high-middle SDI regions; **d** The death cases in the middle SDI regions; **e** The death cases in the low-middle SDI regions; **f** The death cases in the low SDI regions. The three age groups included 15–49 years, 50–69 years, and 70+ years. SDI, socio-demographic index

**Figure S4** The DALYs of CML in three age groups from 1990 to 2017: **a** The DALYs in the globe; **b** The DALYs in the high SDI regions; **c** The DALYs in the high-middle SDI regions; **d** The DALYs in the middle SDI regions; **e** The DALYs in the low-middle SDI regions; **f** The DALYs in the low SDI regions. The three age groups included 15–49 years, 50–69 years, and 70+ years. SDI, socio-demographic index; DALYs, disability-adjusted life years

**Figure S5** The proportion of different ages and sex in CML incidence cases (**a**) and death cases (**b**), and DALYs (c) from 1990 to 2017. DALYs, disability-adjusted life years

**Figure S6** The age-standardized rates of CML DALYs attributed to risk factors from 1990 to 2017 in Global (**a**), High SDI (**b**), High-middle SDI (**c**), Middle SDI (**d**), Low-middle SDI (**e**), Low SDI (**f**). CML, chronic myeloid leukemia; SDI, socio-demographic index. DALYs, disability-adjusted life years

**Figure S7** The percent of CML deaths attributed to risk factors from 1990 to 2017 in Global (**a**), High SDI (**b**), High-middle SDI (**c**), Middle SDI (**d**), Low-middle SDI (**e**), Low SDI (**f**). CML, chronic myeloid leukemia; SDI, socio-demographic index.

**Figure S8** The percent of CML DALYs attributed to risk factors from 1990 to 2017 in Global (**a**), High SDI (**b**), High-middle SDI (**c**), Middle SDI (**d**), Low-middle SDI (**e**), Low SDI (**f**). CML, chronic myeloid leukemia; SDI, socio-demographic index. DALYs, disability-adjusted life years

**Table S1** Top 10 countries or territories with most incidence, death or DALYs cases in 2017.

| Countries | Number of cases in 2017 |
| --- | --- |
| Incidence |  |
| India | 6403.43 |
| China | 2999.47 |
| United States | 1793.68 |
| Germany | 1435.16 |
| Russian Federation | 1200.18 |
| Ethiopia | 1041.62 |
| Italy | 1016.86 |
| Japan | 986.35 |
| France | 958.89 |
| Pakistan | 944.72 |
| Deaths |  |
| India | 5371.36 |
| United States | 1389.62 |
| Germany | 1114.18 |
| China | 1069.83 |
| Ethiopia | 892.84 |
| Russian Federation | 819.59 |
| France | 739.66 |
| Italy | 733.54 |
| Pakistan | 711.17 |
| Indonesia | 590.76 |
| DALYs |  |
| India | 163091.32 |
| Ethiopia | 33234.36 |
| China | 32957.43 |
| United States | 28036.14 |
| Pakistan | 26960.43 |
| Russian Federation | 21143.27 |
| Indonesia | 18718.56 |
| Germany | 18614.87 |
| Brazil | 15263.27 |
| Bangladesh | 14444.31 |

**Table S2** Top 10 countries or territories with highest age-standardized rate of incidence, deaths, or DALYs in 2017.

| Countries | Age-standardized rate in 2017 |
| --- | --- |
| Incidence |  |
| Ethiopia | 1.98 |
| Brunei | 1.72 |
| Honduras | 1.23 |
| Seychelles | 1.2 |
| Denmark | 1.04 |
| Costa Rica | 1.04 |
| Slovenia | 0.96 |
| Austria | 0.93 |
| Grenada | 0.93 |
| Iceland | 0.92 |
| Deaths |  |
| Ethiopia | 1.89 |
| Brunei | 1.09 |
| Honduras | 0.96 |
| Slovenia | 0.84 |
| Costa Rica | 0.82 |
| Seychelles | 0.82 |
| Grenada | 0.75 |
| Afghanistan | 0.74 |
| Haiti | 0.73 |
| Central African Republic | 0.71 |
| DALYs |  |
| Ethiopia | 54.13 |
| Brunei | 33.88 |
| Honduras | 27.25 |
| Afghanistan | 24.16 |
| Grenada | 23.24 |
| Seychelles | 21.9 |
| Central African Republic | 21.36 |
| Haiti | 20.25 |
| Eritrea | 19.87 |
| The Bahamas | 19.22 |

**Table S3** Top 5 countries or territories with the most increase and decrease in age-standardized rate of incidence, deaths, or DALYs from 1990 to 2017.

| Countries | 1990–2017 EAPCs |
| --- | --- |
| Incidence |  |
| Germany | -5.23 |
| United Kingdom | -5.03 |
| Hungary | -5.01 |
| Israel | -4.77 |
| Greenland | -4.49 |
| Philippines | 2.1 |
| Zimbabwe | 2.16 |
| Ecuador | 2.39 |
| El Salvador | 2.71 |
| Jamaica | 2.87 |
| Deaths |  |
| Japan | -5.78 |
| Puerto Rico | -5.73 |
| Finland | -5.62 |
| United Kingdom | -5.54 |
| Singapore | -5.5 |
| Ecuador | 1.67 |
| Lesotho | 1.73 |
| El Salvador | 2.04 |
| Zimbabwe | 2.22 |
| Jamaica | 2.33 |
| DALYs |  |
| Japan | -6.69 |
| United Kingdom | -6.04 |
| Canada | -5.82 |
| United States | -5.78 |
| Hungary | -5.75 |
| Philippines | 1.48 |
| Lesotho | 1.74 |
| El Salvador | 1.93 |
| Zimbabwe | 2.13 |
| Jamaica | 2.6 |


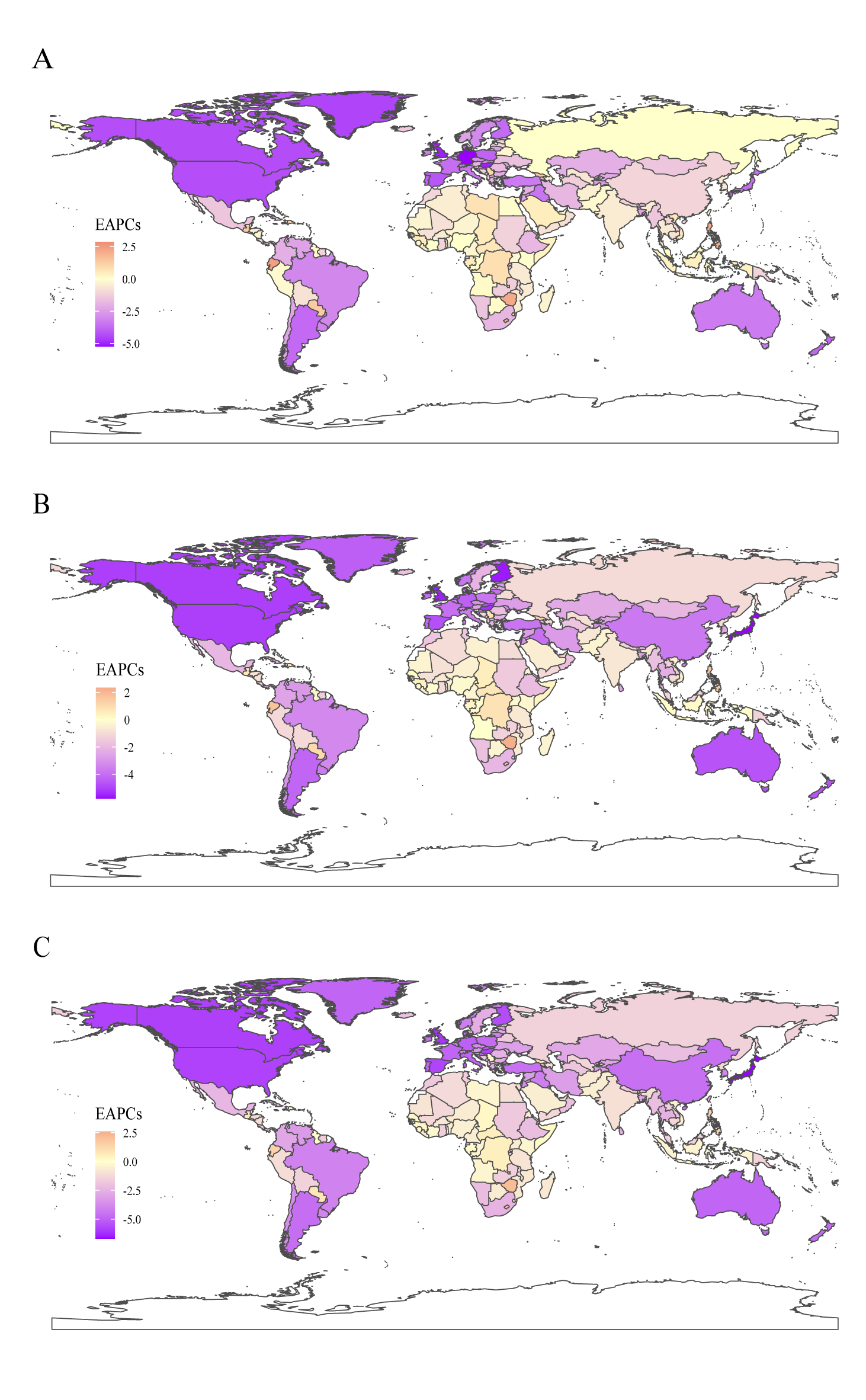


**Figure S1** The global EAPCs of CML in 195 countries or territories in 2017: **a** The EAPCs of incidence in 2017; **b** the EAPCs of deaths in 2017; **c** the EAPCs of DALYs in 2017. CML, chronic myeloid leukemia; DALYs, disability-adjusted life years; EAPCs estimated annual percentage changes


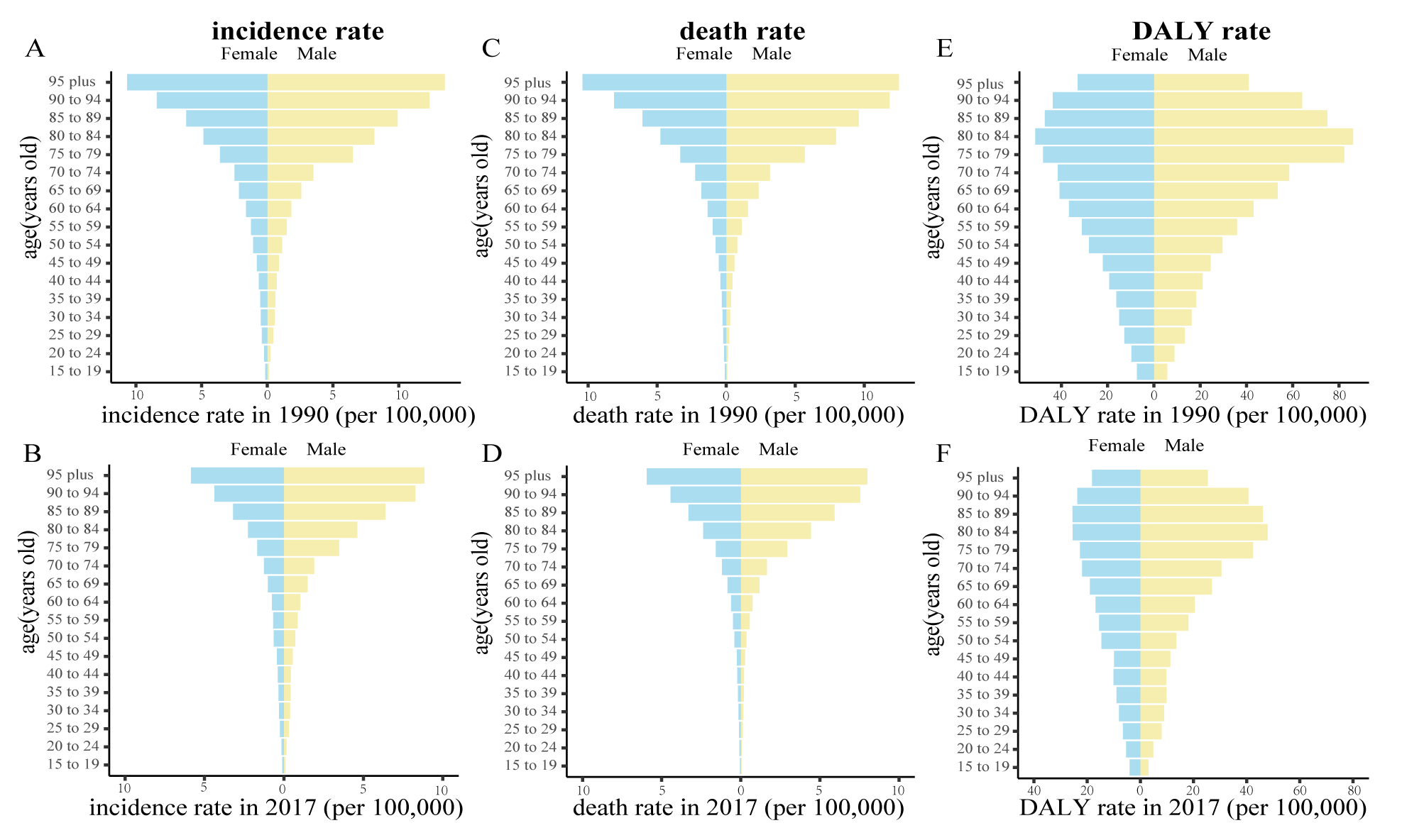


**Figure S2** The incidence, death, and DALY rates of CML in different age groups: **a** The incidence rate in 1990; **b** The incidence rate in 2017; **c** The death rate in 1990; **d** The death rate in 2017; **e** The DALYs rate in 1990; **f** The DALYs rate in 2017. CML, chronic myeloid leukemia; DALYs, disability-adjusted life years


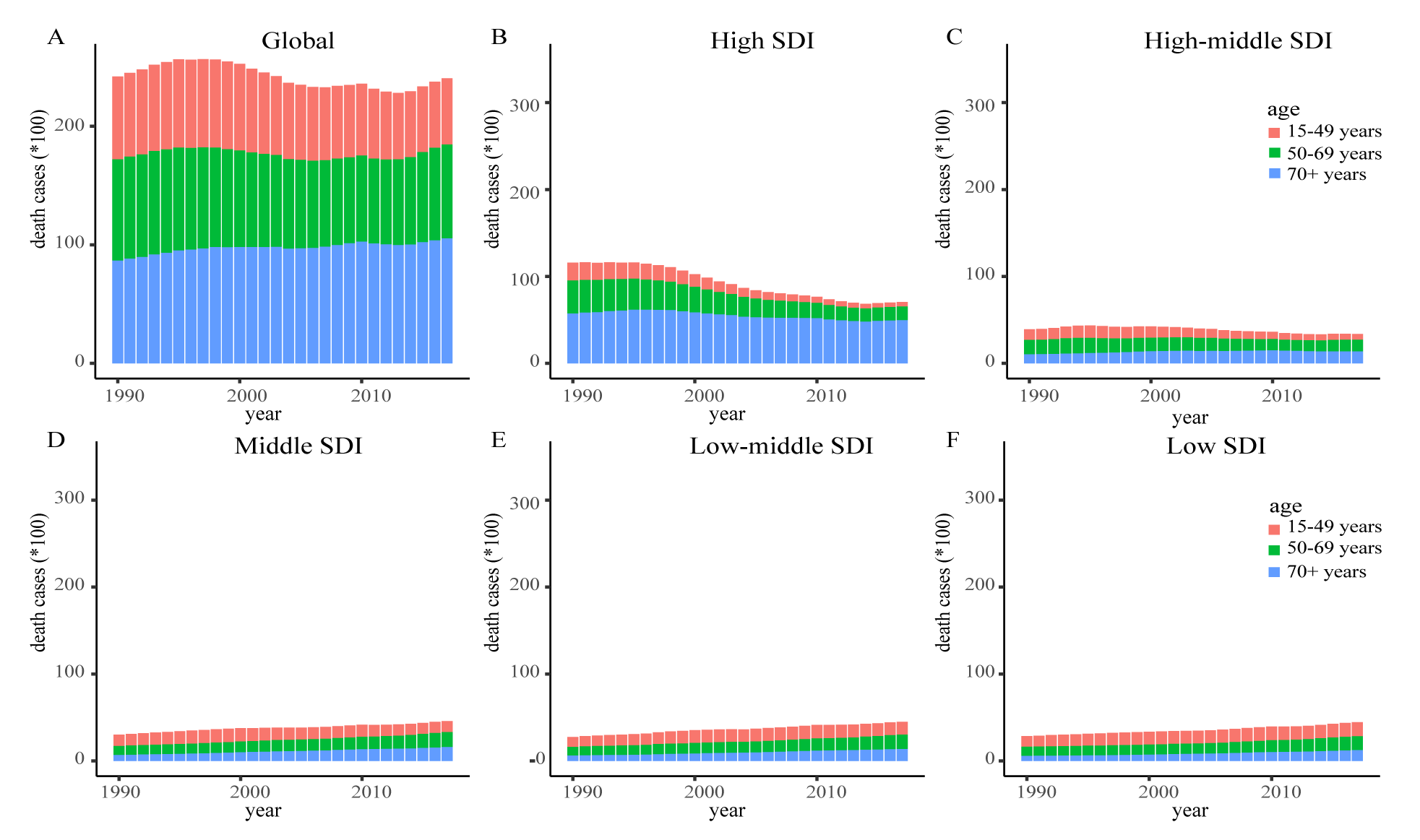


**Figure S3** The death cases of CML in three age groups from 1990 to 2017: **a** The death cases in the globe; **b** The death cases in the high SDI regions; **c** The death cases in the high-middle SDI regions; **d** The death cases in the middle SDI regions; **e** The death cases in the low-middle SDI regions; **f** The death cases in the low SDI regions. The three age groups included 15–49 years, 50–69 years, and 70+ years. SDI, socio-demographic index


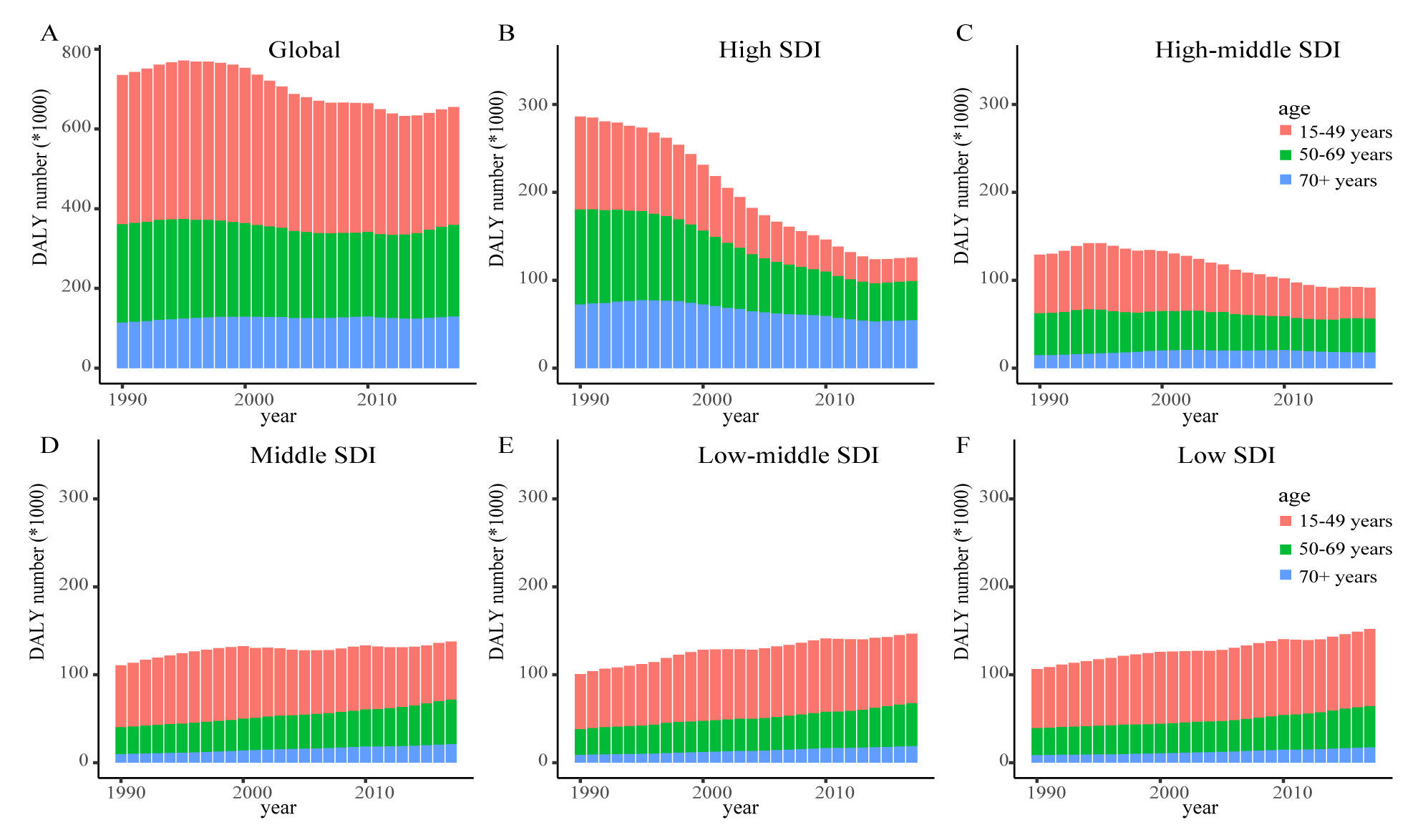


**Figure S4** The DALYs of CML in three age groups from 1990 to 2017: **a** The DALYs in the globe; **b** The DALYs in the high SDI regions; **c** The DALYs in the high-middle SDI regions; **d** The DALYs in the middle SDI regions; **e** The DALYs in the low-middle SDI regions; f The DALYs in the low SDI regions. The three age groups included 15–49 years, 50–69 years, and 70+ years. SDI, socio-demographic index; DALYs, disability-adjusted life years


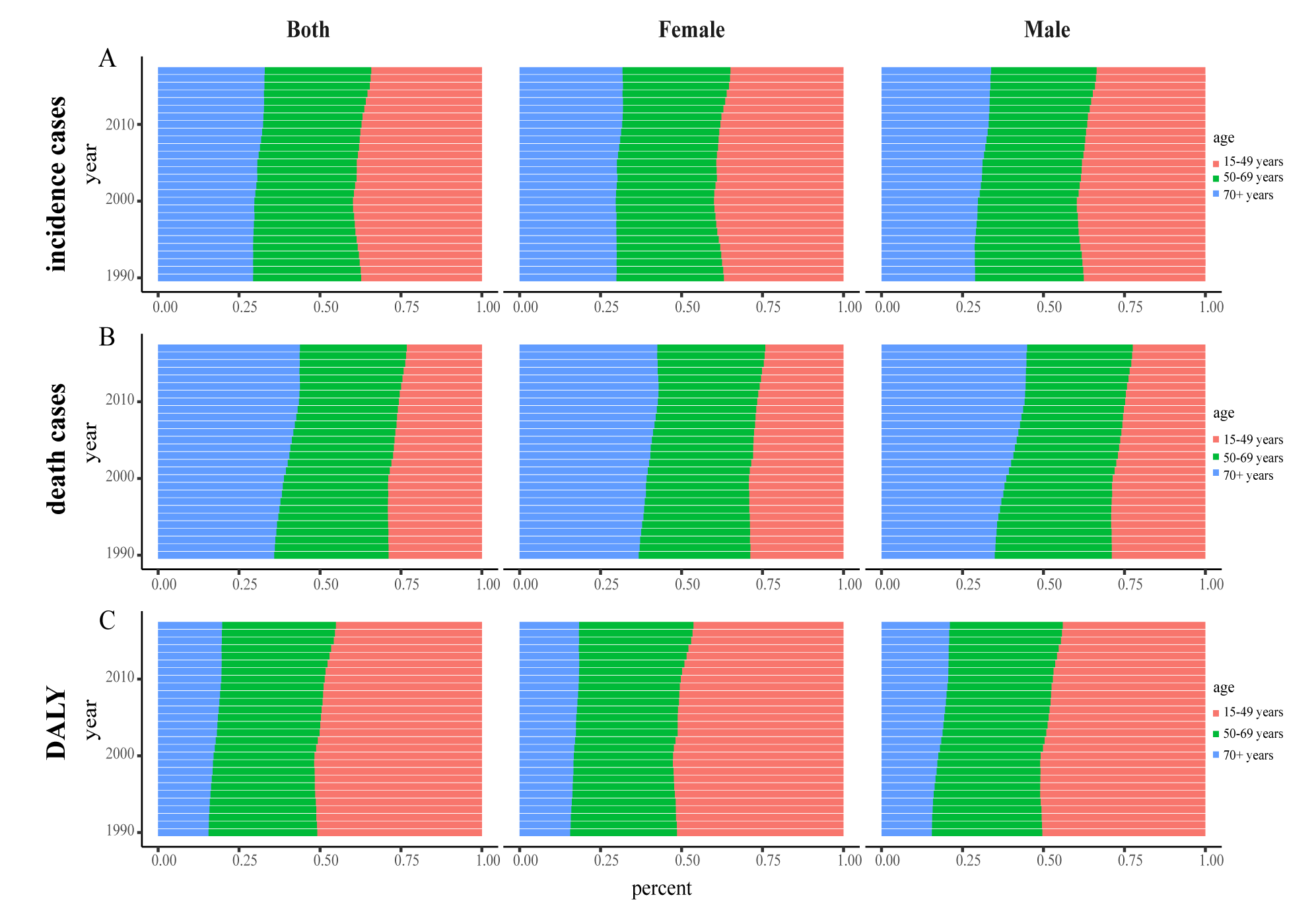


**Figure S5** The proportion of different ages and sex in CML incidence cases (**a**) and death cases (**b**), and DALYs (**c**) from 1990 to 2017. DALYs, disability-adjusted life years


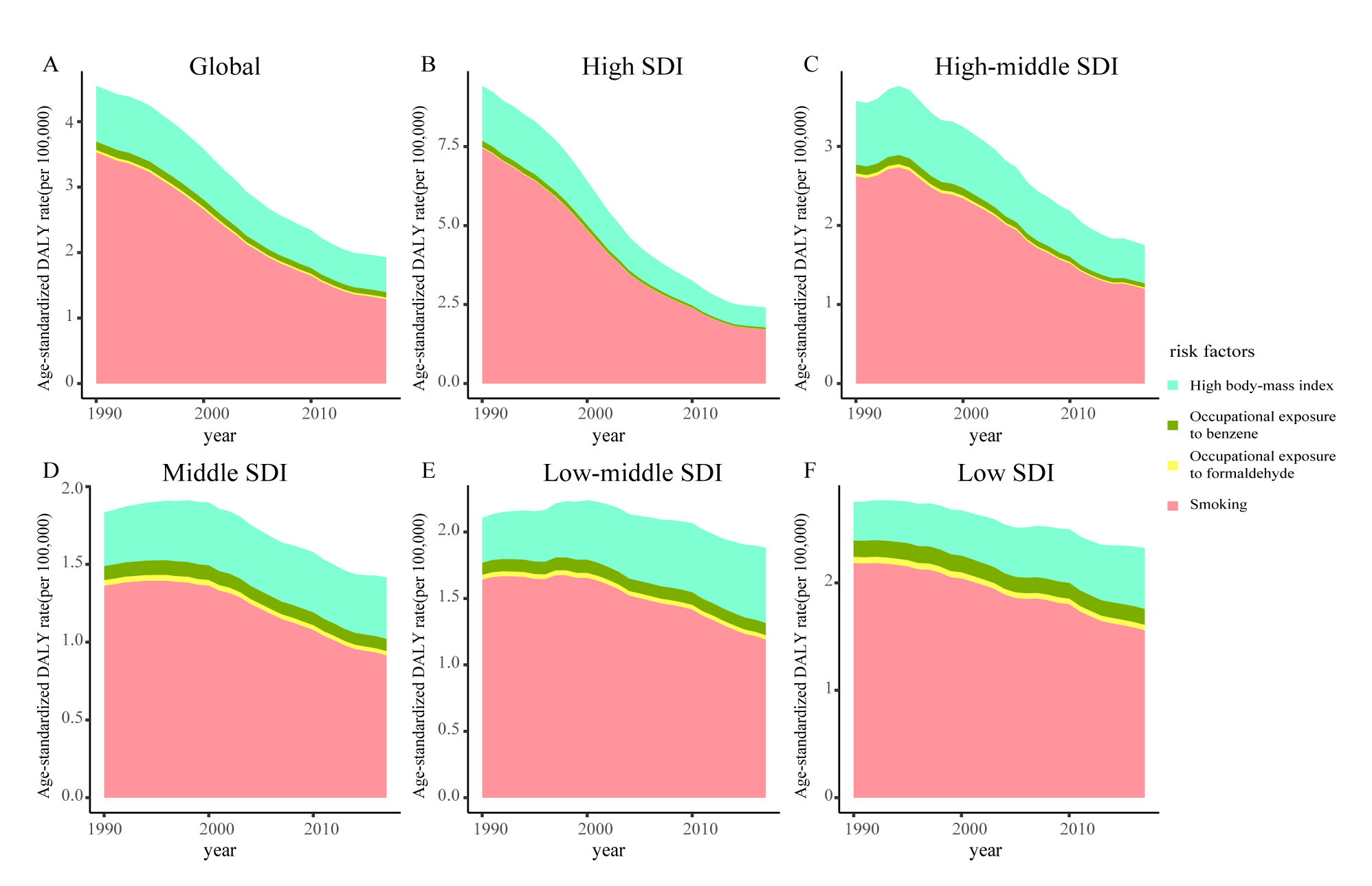


**Figure S6** The age-standardized rates of CML DALYs attributed to risk factors from 1990 to 2017 in Global (**a**), High SDI (**b**), High-middle SDI (**c**), Middle SDI (**d**), Low-middle SDI (**e**), Low SDI (**f**). CML, chronic myeloid leukemia; SDI, socio-demographic index. DALYs, disability-adjusted life years


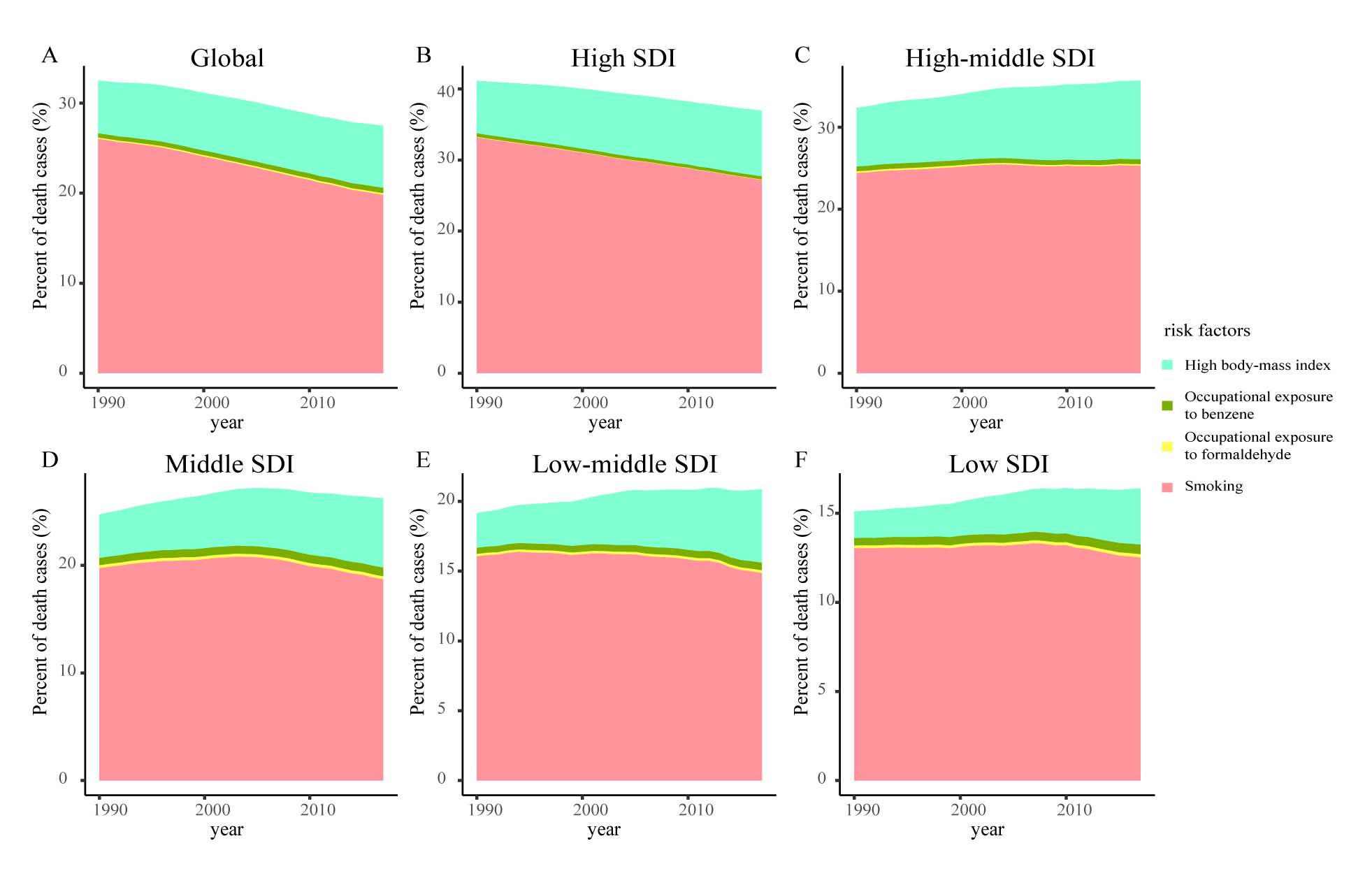


**Figure S7** The percent of CML deaths attributed to risk factors from 1990 to 2017 in Global (**a**), High SDI (**b**), High-middle SDI (**c**), Middle SDI (**d**), Low-middle SDI (**e**), Low SDI (**f**). CML, chronic myeloid leukemia; SDI, socio-demographic index.


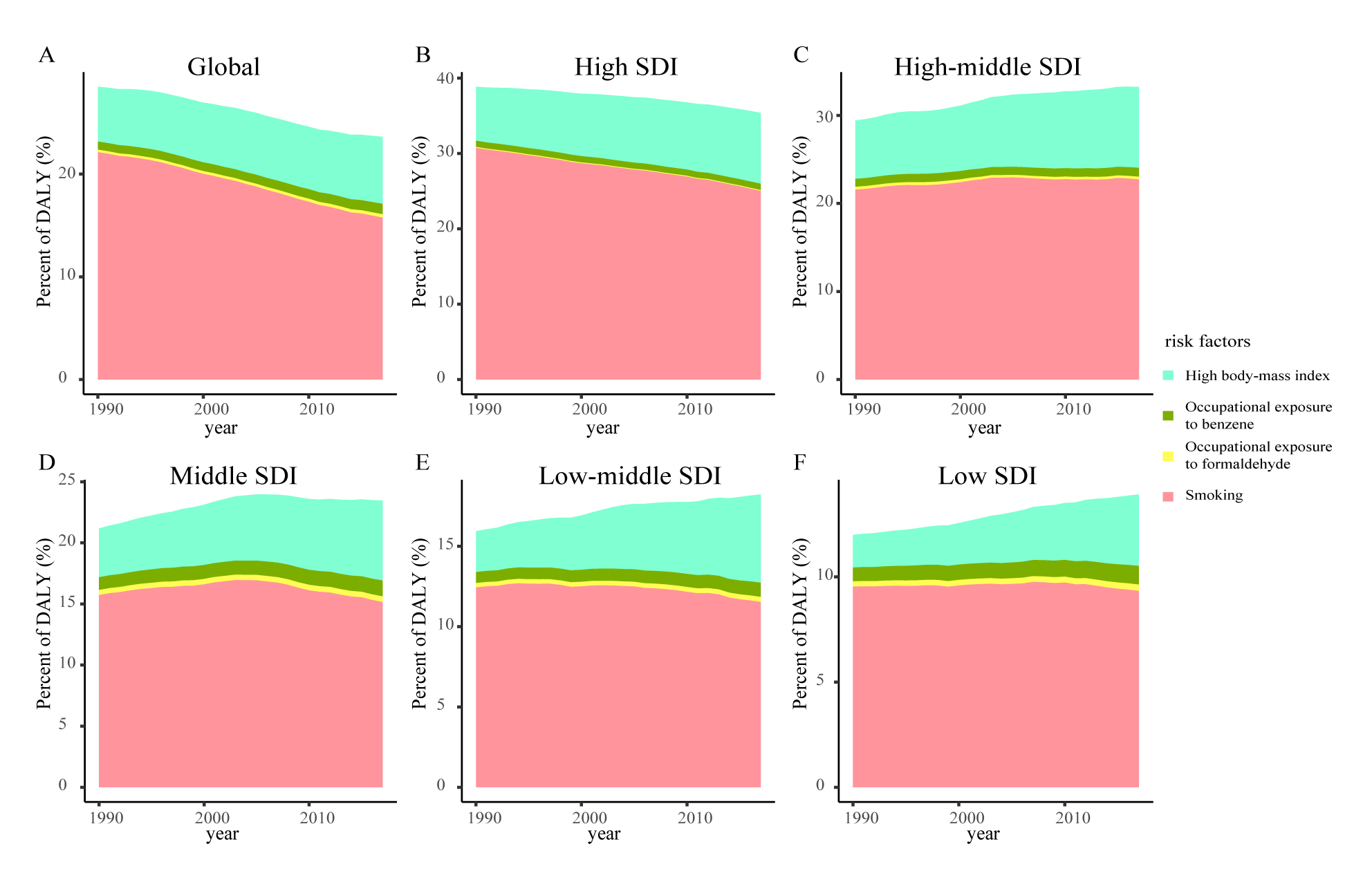


**Figure S8** The percent of CML DALYs attributed to risk factors from 1990 to 2017 in Global (**A**), High SDI (**B**), High-middle SDI (**C**), Middle SDI (**D**), Low-middle SDI (**E**), Low SDI (**F**). CML, chronic myeloid leukemia; SDI, socio-demographic index. DALYs, disability-adjusted life years
